# Untargeted Metabolomic Profiling Reveals Differentially Expressed Serum Metabolites and Pathways in Type 2 Diabetes Patients with and without Cognitive Decline: A Cross-Sectional Study

**DOI:** 10.3390/ijms25042247

**Published:** 2024-02-13

**Authors:** Neyla S. Al-Akl, Olfa Khalifa, Georgios Ponirakis, Aijaz Parray, Marwan Ramadan, Shafi Khan, Mani Chandran, Raheem Ayadathil, Ahmed Elsotouhy, Ahmed Own, Hanadi Al Hamad, Julie Decock, Nehad M. Alajez, Omar Albagha, Rayaz A. Malik, Omar M. A. El-Agnaf, Abdelilah Arredouani

**Affiliations:** 1 Diabetes Research Center, Qatar Biomedical Research Institute (QBRI), Hamad Bin Khalifa University (HBKU), Qatar Foundation (QF), Doha P.O. Box 34110, Qatar; 2 Department of Medicine, Weill Cornell Medicine-Qatar, Qatar Foundation (QF), Doha P.O. Box 24144, Qatar; 3 The Neuroscience Institute, Academic Health System, Hamad Medical Corporation (HMC), Doha P.O. Box 3050, Qatar; 4 Geriatric and Memory Clinic, Rumailah Hospital, Hamad Medical Corporation (HMC), Doha P.O. Box 3050, Qatar; 5 Department of Clinical Radiology, Weill Cornell Medicine-Qatar, Qatar Foundation, Doha P.O. Box 24144, Qatar; 6 Neuroradiology Department, Hamad General Hospital, Hamad Medical Corporation, Doha P.O. Box 3050, Qatar; 7 College of Health and Life Sciences (CHLS), Hamad Bin Khalifa University (HBKU), Qatar Foundation (QF), Doha P.O. Box 34110, Qatar; 8 Cancer Research Center, Qatar Biomedical Research Institute (QBRI), Hamad Bin Khalifa University (HBKU), Qatar Foundation (QF), Doha P.O. Box 34110, Qatar; 9 Neurological Disorders Research Center, Qatar Biomedical Research Institute (QBRI), Hamad Bin Khalifa University (HBKU), Qatar Foundation (QF), Doha P.O. Box 34110, Qatar

**Keywords:** dementia, mild cognitive impairment, type 2 diabetes, metabolomics, diabetes-associated cognitive decline

## Abstract

Diabetes is recognized as a risk factor for cognitive decline, but the underlying mechanisms remain elusive. We aimed to identify the metabolic pathways altered in diabetes-associated cognitive decline (DACD) using untargeted metabolomics. We conducted liquid chromatography–mass spectrometry-based untargeted metabolomics to profile serum metabolite levels in 100 patients with type 2 diabetes (T2D) (54 without and 46 with DACD). Multivariate statistical tools were used to identify the differentially expressed metabolites (DEMs), and enrichment and pathways analyses were used to identify the signaling pathways associated with the DEMs. The receiver operating characteristic (ROC) analysis was employed to assess the diagnostic accuracy of a set of metabolites. We identified twenty DEMs, seven up- and thirteen downregulated in the DACD vs. DM group. Chemometric analysis revealed distinct clustering between the two groups. Metabolite set enrichment analysis found significant enrichment in various metabolite sets, including galactose metabolism, arginine and unsaturated fatty acid biosynthesis, citrate cycle, fructose and mannose, alanine, aspartate, and glutamate metabolism. Pathway analysis identified six significantly altered pathways, including arginine and unsaturated fatty acid biosynthesis, and the metabolism of the citrate cycle, alanine, aspartate, glutamate, a-linolenic acid, and glycerophospholipids. Classifier models with AUC-ROC > 90% were developed using individual metabolites or a combination of individual metabolites and metabolite ratios. Our study provides evidence of perturbations in multiple metabolic pathways in patients with DACD. The distinct DEMs identified in this study hold promise as diagnostic biomarkers for DACD patients.

## 1. Introduction

The most recent analysis of the Global Burden of Disease has projected a significant rise in the prevalence of diabetes, with an estimated 1.3 billion individuals worldwide expected to have diabetes by 2050, compared to 529 million in 2021 [1]. Diabetes is known to be associated with an increased risk of dementia, including Alzheimer’s disease (AD), vascular dementia (VD), and mild cognitive impairment (MCI). Indeed, long-term studies have demonstrated that the incidence of dementia is approximately 80% higher in individuals with diabetes than in those without diabetes in a 25-year follow-up study conducted in the USA [2]. A comprehensive meta-analysis of prospective studies involving over 9 million individuals revealed that both prediabetes (RR: 1.18, 95% CI: 1.02–1.36) and diabetes (1.25- to 1.91-fold) conferred an excess risk for dementia [3]. Similarly, another meta-analysis of 19 population-based studies reported an increased relative risk for MCI in subjects with diabetes (RR of 1.21; 95% CI 1.02–1.45) [4].

Moreover, a large-scale cohort study involving over 150,000 nondemented adults enrolled at baseline before 65 years found that diabetes was associated with a higher risk of developing dementia during follow-up [5]. Diabetes also accelerates the progression from MCI to dementia [6], and individuals with diabetes experience a 24% faster cognitive decline than those without diabetes, which was associated with diabetes duration and glycemic control [7]. Notably, the age of diabetes onset has been identified as a significant risk factor for dementia, with earlier onset associated with a higher risk of dementia [8].

Even newly diagnosed diabetes has been found to increase the risk of dementia (HR 1.16 (CI 95% 1.15–1.18)), highlighting the importance of considering diabetes in the early stages of the disease [9]. Individuals with prediabetes are also at increased risk of dementia [10].

Despite the well-established association between diabetes and cognitive decline and numerous investigations, the exact underlying pathophysiological mechanisms of diabetes-associated cognitive decline (DACD) remain elusive. Various markers have been associated with DACD, including hyperinsulinemia, hypoglycemia, hyperglycemia, hypertension, diabetic retinal microangiopathy, diabetic renal function impairment, dyslipidemia, age, T2D duration, smoking, obesity, hearing loss, sedentary, depression, low educational attainment, and genetic factors (reviewed in [11]). However, the results from different studies are inconsistent. It is likely that in the majority of T2D patients, multiple factors determine the cognitive decline.

While cognitive decline biomarker assessments include both cerebrospinal fluid analysis and advanced neuroimaging modalities, non-invasive blood-based biomarkers hold the most promise. There has been an increase in the number of DACD blood biomarker studies performed over the past years [11,12], yet there remains a paucity of clinically meaningful diagnostic blood biomarkers.

Thus, several prior studies have investigated the links between circulating factors and cognitive impairment in T2D patients. For instance, higher plasma levels of Nicotinamide phosphoribosyltransferase and adipsin were associated with MCI in Chinese T2D patients [13,14]. Furthermore, Wang et al. reported a significant correlation between high levels of plasminogen activator inhibitor 1 (PAI-1) and a low tissue plasminogen activator (tPA)/PAI-1 molar ratio with DACD, particularly affecting memory function [15]. Moreover, cognitive susceptibility, notably attention and execution deficits, has been observed in T2D patients with elevated plasma levels of free fatty acids (FFAs) [16]. Associations between inflammatory factors and cognitive decline in diabetes have also been explored. Thus, Marioni et al. explored the circulating inflammation markers CRP, IL-6, and TNF-α in 1066 patients with diabetes, demonstrating that IL-6 and TNF-α were associated with cognitive function [17]. Increased levels of CRP, IL-6, and TNF-α were also revealed in patients with both diabetes and MCI [18,19]. IL-1β was also increased in patients with MCI subjects when compared with normal controls [20]. Other studies have shown a link between increased levels of renal markers, such as the albumin/creatinine ratio (ACR) and cystatin C, and greater decreases in cognitive tests in patients with diabetes [12,21]. Other studies suggested that the serum brain-derived neurotrophic factor (BDNF) may play a role in the pathophysiology of cognitive deficits, especially delayed memory in T2D patients [22,23].

In the pursuit of more precise identification of high-risk individuals, transcending conventional demographic and laboratory parameters is imperative, necessitating the exploration of innovative approaches. Over the past two decades, a proliferation of studies have employed omics technologies, including proteomics, metabolomics, and genomics, to discern metabolic and signaling aberrations, as well as blood-based biomarkers, across various disorders. Remarkably, metabolomics, a discipline centered on the study of metabolite profiles, has emerged as a pivotal instrument in comprehending the pathophysiological underpinnings of diverse diseases. Its application has markedly enhanced the capabilities for diagnosing, prognosticating, and treating numerous ailments [24,25].

Numerous metabolomics investigations have explored DACD within animal models, employing various biological specimens including brain tissues, cerebrospinal fluid (CSF), blood, or urine samples [26,27,28,29,30]. However, the utilization of human brain tissue or CSF is fraught with impracticality due to the postmortem nature of brain tissue acquisition and the highly invasive procedure associated with CSF collection. Consequently, researchers have redirected their focus towards the use of peripheral blood as a more accessible alternative. For instance, Zhang et al. conducted a comprehensive analysis of metabolic alterations associated with DACD in humans, utilizing plasma samples and metabonomics techniques [31]. Their investigation unveiled significant disruptions in sphingolipids metabolism, bile acids metabolism, and the uric acid metabolism pathway, shedding light on the intricate metabolic dysregulations observed in both T2D and DACD. In a complementary endeavor, Morris and collaborators utilized metabolomics methodologies alongside serum samples to elucidate the relationship between oxygenated lipids and DACD [32]. Furthermore, Sun and colleagues undertook an integrative approach, combining blood samples with metabolomics profiling to identify risk factors contributing to cognitive impairment in patients with T2D [33]. Their collective findings highlighted the substantive association between elevated levels of glutamate, phenylalanine, tyrosine, proline, and homocysteine, coupled with a diminished level of glutamine, and cognitive impairment in individuals with T2D patients. Despite the limited number of studies, their findings underscore the significance of peripheral blood as a viable matrix for unraveling the intricate metabolic signatures implicated in DACD, thereby advancing our understanding of the pathophysiological mechanisms underlying cognitive decline in the context of diabetes mellitus.

The research presented in this paper was undertaken by a multidisciplinary team to fill a gap in our knowledge of the mechanisms underlying the increased risk of dementia, including Alzheimer’s disease (AD), vascular dementia (VD), and mild cognitive impairment (MCI), in individuals with T2D. Our study methodology uses a comprehensive profiling of serum metabolites, which has allowed us to obtain a robust and comprehensive representation of the altered metabolic pathways in subjects with DACD.

## 2. Results

### 2.1. Characteristics of the Study Population

The study population consisted of 100 subjects, 42% of whom were women. Table 1 provides a detailed overview of the clinical and laboratory characteristics of the participants. Of these subjects with T2D, 54 had normal cognitive function (the DM group), while 46 displayed cognitive impairment with either MCI or dementia, hereafter referred to as the diabetes-associated cognitive decline (DACD) group. The DACD group had a significantly lower MoCA score (29 ± 1 vs. 21 ± 8, *p* < 0.001). Additionally, the DACD group exhibited lower FIM scores (*p* = 0.03) than the DM group. The individuals with DACD were also significantly older (*p* < 0.001) and had a higher creatinine level (*p* = 0.004) and MCV (*p* = 0.003). However, there were no notable differences between the groups regarding BMI, blood pressure, Hb, thyroid function, B_12_, or lipid profile, except for a slightly lower HbA_1c_ level in the DACD group (*p* < 0.04).

### 2.2. Identification of Differentially Expressed Metabolites between DM and DACD Samples

**Figure 1:**
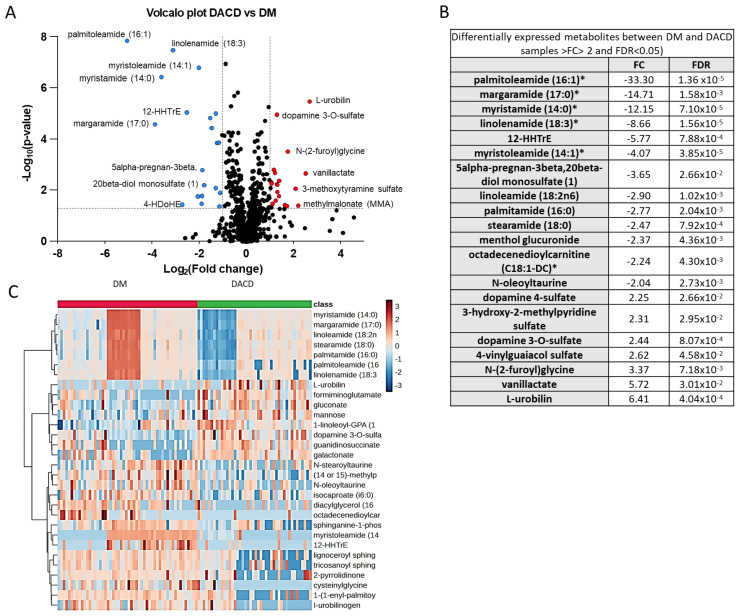
Differential expression of the plasma metabolome between the DACD and DM groups. (**A**) Volcano plot displaying the log2-fold change (*x*-axis) against the −log10 statistical *p*-value (*y*-axis) for all metabolites. Downregulated and upregulated metabolites in DACD (*p* < 0.05 and FC > 2) are shown in blue and red, respectively. Nonsignificant metabolites between the two groups are in black. (**B**) List of the differentially expressed metabolites with their FC and FDR values. (**C**) Supervised cluster analysis across the DM and the DACD groups using the top 30 differentially expressed metabolites. The blue color represents downregulated metabolites, while the brown color represents upregulated metabolites in the DACD group. * Denotes a compound whose identity hasn’t been officially verified using a standard, yet we possess a high level of confidence in its identification.

The untargeted metabolomic profiling conducted in this study identified 1223 metabolites, of which 251 were unnamed. Our analysis only focused on the 972 named metabolites (Supplementary Table S1). Among these metabolites, more than half (617) belong to the amino acid (211) and lipid (406) super pathways. Approximately one-fifth (187) are xenobiotics, while the rest belong to carbohydrate (22), cofactors and vitamins (31), energy (10), nucleotide (39), and peptide (44) super pathways or are partially characterized molecules (22). The volcano plot in Figure 1A indicates that using FC > 2 and FDR < 0.05, 20 metabolites were differentially expressed between the DM and DACD groups. Among these, thirteen were downregulated and seven were upregulated in the DACD vs. DM group. The list of 20 metabolites with >2-fold change and FDR < 0.05 is shown in Figure 1B. Furthermore, the heatmap in Figure 1C compares the metabolite profiles of the two groups, highlighting the top thirty metabolites.

### 2.3. Chemometric Analysis

**Figure 2:**
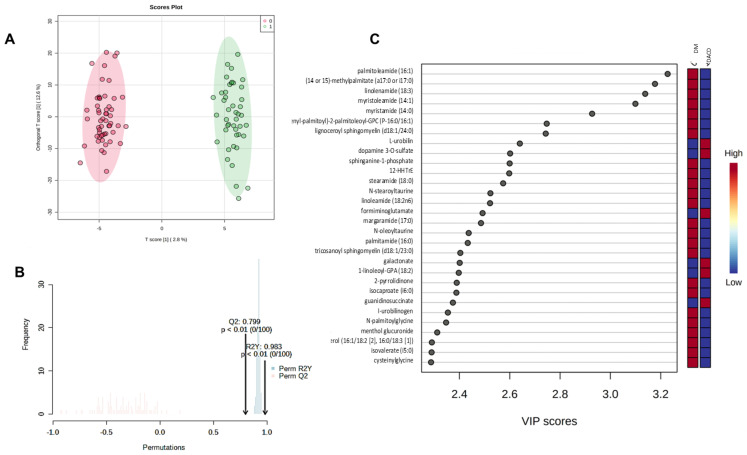
Chemometric analysis of metabolomic data sets of the DM and DACD samples. (**A**) Score plot of the orthogonal partial least squares discriminant analysis (OPLSDA) model. The pink and green dots represent the DM and DACD samples, respectively. (**B**) Results of the 1000-time permutation test of the OPLSDA model; the empirical *p*-values for R2Y and Q2 were all <0.001. (**C**) Top 30 metabolites important for the separation between the DM and DACD samples based on the VIP score. Red and blue colors on the scale on the right indicate upregulation and downregulation in the respective group.

In addition to the differential expression analysis, we performed chemometric analysis on the data sets. PCA detected the presence of two outliers that were excluded from further analysis. We then applied OPLSDA to examine the clustering of the two groups. The score plot in Figure 2A clearly shows that the DM and DACD samples could be successfully distinguished from each other. The parameters of the OPLSDA model (R2Y (cum) = 0.983, Q2 (cum) = 0.799) calculated by 10-fold cross-validation also suggested that the model had satisfactory robustness. The empirical *p*-values for R2Y and Q2 in the validation of this OPLSDA model based on a 1000-time permutation test were all <0.001 (Figure 2B), indicating that the observed statistic is not part of the distribution formed by those from the permuted data, implying that the OPLS-DA model can successfully detect markers differentiating between DM and DACD.

We then used the variable importance in projection (VIP) score to identify the essential metabolites for the separation observed in the OPLS-DA score plot. We identified 271 metabolites with a VIP > 1 (the threshold that is typically used to select relevant features) (Supplementary Table S2). Figure 2C shows the top 30 features that contribute the most to the separation seen in Figure 2A.

### 2.4. Metabolic Pathway Analysis

**Figure 3:**
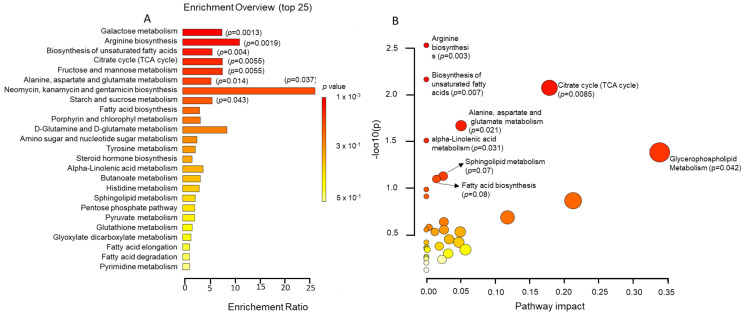
The results of pathway analysis and enrichment analysis of the metabolomic data. (**A**) Enrichment analysis based on the Small Molecule Pathway Database (SMPDB). (**B**) Pathway analysis based on the KEGG database.

For a better understanding of the metabolic dysregulation that may underlie the development of DACD, we used all the metabolites with VIP > 1 to perform metabolite set enrichment (Figure 3A) and metabolic pathway analyses (Figure 3B) using the KEGG database. We were mainly interested in the endogenous metabolites that could drive systemic biological processes, so we excluded 50 metabolites identified as xenobiotics.

Metabolite set enrichment analysis revealed eight metabolite sets that were significantly enriched (*p* < 0.05), of which five had an FDR < 0.1, including galactose metabolism (*p* = 0.0013, FDR = 0.08), arginine biosynthesis (*p* = 0.00019, FDR = 0.08), the biosynthesis of unsaturated fatty acids (*p* = 0.0039, FDR = 0.09), citrate cycle (TCA cycle) (*p* = 0.0055, FDR = 0.09), fructose and mannose metabolism (*p* = 0.0055, FDR = 0.09), alanine, aspartate and glutamate metabolism (*p* = 0.014), neomycin, kanamycin and gentamicin biosynthesis (*p* = 0.037), and starch and sucrose metabolism (*p* = 0.043). On the other hand, pathway analysis yielded six significantly (*p* < 0.05) altered pathways, including arginine biosynthesis (*p* = 0.003), the biosynthesis of unsaturated fatty acids (*p* = 0.007), citrate cycle (TCA cycle) (*p* = 0.0085), alanine, aspartate, and glutamate metabolism (*p* = 0.021), alpha-linolenic acid metabolism (*p* = 0.031), and glycerophospholipid metabolism (*p* = 0.042). Two pathways had a *p*-value <0.1: sphingolipid metabolism (*p* = 0.07) and fatty acid biosynthesis (*p* = 0.08).

### 2.5. Biomarker Discovery

To evaluate the diagnostic efficacy of the differentially expressed metabolites, the criteria we used consisted of FC > 1.5, FDR < 0.05, and VIP > 2. Among the initial pool of metabolites, 50 met these criteria. However, six of these metabolites were xenobiotics and were excluded from further analysis. Thus, 44 metabolites, 13 upregulated and 31 downregulated in the DACD group compared to the DM group, remained for further evaluation (Supplementary Table S3).

Diagnostic utility was assessed by constructing ROC curves and calculating AUCs based on multivariate logistic regression analysis with internal tenfold cross-validation and adjustment for age and sex. Supplementary Table S4 displays the adjusted AUC, specificity, sensitivity, odds ratios, and *p*-values of individual metabolites. Only metabolites with a significant *p*-value (<0.05) in the logistic regression model are shown. The discriminatory accuracy of all the individual metabolites in Table S4 exceeded 80%.

**Figure 4:**
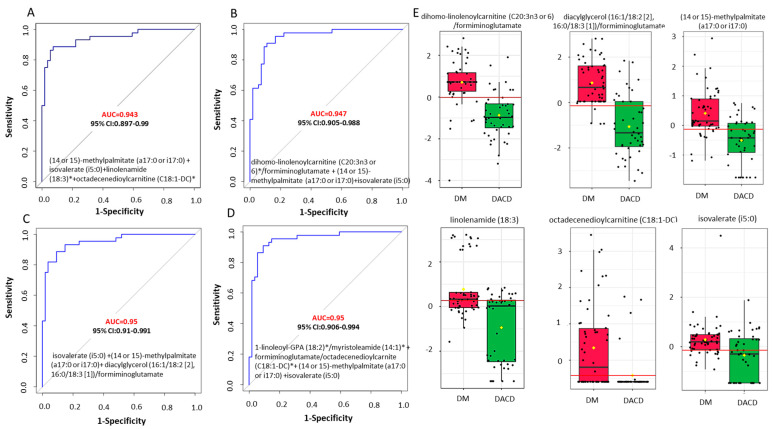
The ROC curves generated in the Biomarker Module of Metaboanalyst. (**A**–**D**) The top four biomarker candidate metabolites and metabolite ratios identified based on ROC curve analysis performed with 50 serum metabolic features, and their ratios estimated for their relative concentrations in the DM and DACD study groups. The computed AUC-ROC, 95% confidence interval (CI), and marker metabolites are shown for each model. The AUC-ROC is shown in red to highlight the diagnostic potential of the model. (**E**) The whisker plots shown on the right revealed significantly decreased serum levels of these metabolites and their ratios in the DACD (green) group compared to the DM (red) group. The boxes denote interquartile ranges, the average value are represented by the horizontal black line, and yellow diamond representing median (or 50th percentile value) within each box. The bottom and top boundaries of the boxes are the 25th and 75th percentiles, respectively. The lower and upper whiskers are the 5th and 95th percentiles, respectively. Outlier points beyond this range are indicated above or below the whiskers. Each box plot shows quantitative variations in metabolic concentrations. * Denotes a compound whose identity hasn’t been officially verified using a standard, yet we possess a high level of confidence in its identification. DACD: diabetes-associated cognitive dysfunction.

To further improve the diagnostic accuracy of the metabolites, we constructed models based on multiple metabolites. The combination of four metabolites, (14 or 15)-methyl palmitate (a17:0 or i17:0), isovalerate (i5:0), linolenamide (18:3), and octadecenedioylcarnitine (C18:1-DC), yielded the best discriminatory accuracy. The sex- and age-adjusted AUC of this model was 0.943 (95% CI: 0.896~0.990), with a sensitivity of 0.886 (95% CI: 0.886~0.980) and a specificity of 0.926 (95% CI: 0.856~0.99) (Figure 4A).

Afterwards, we employed metabolite ratios to boost discriminating efficacy even further, as ratios between two metabolite concentrations may carry more information than the two corresponding metabolite concentrations alone. We calculated all the possible metabolite pairs and then examined the discriminatory efficacy of the top-ranked ratios (based on their respective AUC values) after adjusting for age and sex. None of the metabolite ratios could improve accuracy beyond 0.943. However, the combinations of metabolite ratios with two individual metabolites (isovalerate (i5:0) and (14 or 15)-methylpalmitate (a17:0 or i17:0)) slightly increased the accuracy to 0.95 (Figure 4D).

## 3. Discussion

In this study, we combined unbiased untargeted serum metabolomic profiling with multivariate statistical analysis to uncover metabolic alterations in diabetic patients with and without cognitive impairment. Our analysis identified important metabolites that exhibited disease-specific patterns in diabetic patients with and without DACD. Some of these metabolites are implicated in key metabolic pathways, including the tricarboxylic acid cycle (TCA) and the metabolism of amino acids, fatty acids, and carbohydrates.

While it is established that DM increases the risk of cognitive decline and that DACD is recognized as one of the chronic complications of DM [34,35,36,37,38,39,40,41], the mechanisms underlying cognitive decline in diabetic patients remain elusive. Despite hyperglycemia being a major risk factor for diabetes complications, previous randomized controlled intervention studies have found little evidence that stringent glycemic control improves cognitive performance [42]. In fact, there is a strong link between hypoglycemic episodes and increased dementia risk [9,43,44,45].

Our enrichment analysis revealed that galactose metabolism was one of the most enriched metabolic pathways, with four metabolites (glucose, fructose, mannose, and sorbitol) showing significant abundance in patients with DACD. Elevated sorbitol levels in various tissues of rats with streptozotocin-induced diabetes were observed [46], and these elevated levels have been associated with vascular damage and nerve degeneration [47]. In investigations of AD patients, elevated glucose, sorbitol, and fructose levels have been observed in AD brain regions compared to controls [48]. Some studies have suggested a potential link between high-fructose diets and cognitive impairment, indicating that long-term fructose consumption can lead to insulin resistance in the brain, contributing to cognitive deficits [49]. Excessive fructose intake has been associated with oxidative stress and inflammation, both of which are implicated in AD [50].

Furthermore, our findings indicate that DACD is associated with alterations in arginine biosynthesis and metabolism of the amino acids alanine, aspartate, and glutamate. Among the metabolites involved in these pathways, we observed significantly higher levels of three metabolites (N-acetylornithine, alpha-ketoglutarate (AKG), and fumarate) in patients with DACD. AKG is critical in various metabolic pathways, including the TCA cycle, amino acid anabolic and catabolic processes, and collagen formation. Recent research has highlighted the key position of AKG as a significant signaling hub with antiaging and neuroprotective effects, although the mechanisms underlying its action remain elusive. AKG can enter the brain through different mechanisms, including carrier-mediated processes, simple diffusion, and transport across mitochondrial membranes.

Furthermore, AKG is involved in various signaling pathways in cellular redox control, epigenetic processes, and the inflammatory response [51]. Recent discoveries in metabolic pathway modulation point to the key position of AKG as a significant signaling hub, emphasizing its antiaging and neuroprotective effects, although the mechanisms underlying its action remain elusive [52]. AKG can enter the brain through different mechanisms, including carrier-mediated processes, simple diffusion, and transport across mitochondrial membranes [53,54]. It plays a crucial role in neurotransmission, memory, and protection against oxidative stress by detoxifying ammonia in the brain and reducing oxidative stress levels. Increased oxidative stress is associated with neurological diseases, such as AD, Parkinson’s disease (PD), Huntington’s disease, and amyotrophic lateral sclerosis (ALS) [55]. AKG can protect the brain from oxidative injury by boosting antioxidative enzyme concentrations and scavenging reactive oxygen species in neurons [55]. The reasons for the high levels of AKG in patients with DACD in our cohort warrant further investigation; AKG may be a defense mechanism to protect against further neuronal damage. This observation raises the question of whether AKG supplementation in diabetic patients may reduce the risk of dementia.

A noteworthy finding in our study was the significantly elevated levels of N-acetylornithine in individuals with DACD. N-acetylornithine, an intermediary metabolite in the arginine metabolic pathway, has been implicated in chronic kidney disease and is considered a promising biomarker for assessing human kidney function. A higher concentration of N-acetylornithine correlated with a reduced estimated glomerular filtration rate [56]. No previous scientific investigation has established a connection between N-acetylornithine and cognitive decline. However, a study on mice treated with haloperidol, an antipsychotic used for schizophrenia, reported variations in N-acetylornithine levels [57]. Furthermore, a magnetic resonance-based study focusing on blood metabolites in humans demonstrated lower N-acetylornithine levels in individuals with schizophrenia [58]. Interestingly, N-acetylornithine was found to be significantly higher in early-stage AD compared with MCI patients [59]. Further investigations are warranted to gain a better understanding of the potential role of this metabolite in DACD.

The biosynthesis of unsaturated fatty acids was also a significant metabolite-enriched set. Among the 36 metabolites within this particular pathway, the serum concentrations of four specific metabolites, namely, palmitic acid, gamma-linolenic acid, docosahexaenoic acid (DHA), and eicosapentaenoic acid (EPA), exhibited a significant decrease in individuals with DACD when compared to the DM cohort. This finding is in line with previous studies that suggested that high levels of monosaturated and polyunsaturated fatty acids had protective effects from age-related cognitive decline [60,61,62].

EPA and DHA exhibit distinct biological effects. DHA, a prominent constituent of polyunsaturated fatty acids in the brain, facilitates neuroplasticity and neuroprotection. Conversely, EPA is scarcely present in the brain but modulates inflammatory responses and immune function [60]. A recent prospective study reported that higher concentrations of EPA were associated with a lower incidence of AD and a decreased risk for all-cause dementia and AD among apolipoprotein Eε4 (APOE ε4) noncarriers but not among APOEε4 carriers [61]. Other fatty acids, including docosahexaenoic acid (DHA), alpha-linolenic acid (ALA), linoleic acid (LA), dihomoγ-linolenic acid (DGLA), and arachidonic acid (AA), have shown no significant association with AD or dementia. In a two-year follow-up study, Chu and colleagues reported that a higher serum DHA was associated with a slower cognitive decline in patients with AD [60]. A recent meta-analysis of prospective cohort studies showed that each 0.1 g/day increase in DHA or EPA was associated with an 8–9.9% lower risk of cognitive decline [62]. A ten-year follow-up study among community-dwelling older adults in Japan demonstrated that a higher level of serum DHA may prevent cognitive decline [63]. In male streptozotocin (STZ) diabetic rats, a two-week treatment with the soluble epoxide hydrolase (sEH) inhibitor N-[1-(1-oxopropyl)-4-piperidinyl]-N′-[4-(trifluoromethoxy)phenyl)-urea (TPPU) (1 mg/kg) + DHA (100 mg/kg) increased the memory response of diabetic rats compared to untreated diabetic rats [64]. The treatment reduced oxidative stress, minimized inflammation in the brains of diabetic rats, and significantly protected neurons against neuronal damage.

Pathway analysis revealed enrichment in the citrate cycle with a higher citrate level in patients with DACD. A recent metabolomic profiling study involving 110,655 people from the UK biobank found that citrate was positively associated with dementia [65], increased brain and hippocampal atrophy, and white matter hyperintensities, which are associated with AD and dementia [66].

Our investigation revealed a noteworthy enrichment of pathways related to alanine, aspartate, and glutamate metabolism, underscoring the potential significance of these amino acids in exerting deleterious effects on cognitive processes. Recent investigations have underscored the pivotal significance of glucose metabolism in the biosynthesis of neurotransmitters and neuromodulators, such as glutamate, GABA, and others [67]. Therefore, impairments in glucose metabolism can engender disruptions in the synthesis of these essential molecules, potentially contributing to neuronal dysfunction. A pertinent illustration of this phenomenon is evident in diabetic mice during the ninth week of disease progression [68]. At this point, substantial pathological damage had already manifested in the hippocampal region. Notably, discernible reductions were observed in neurotransmitter levels, encompassing glutamate, glutamine, and aspartate, as well as in amino acids, such as valine, leucine, isoleucine, taurine, succinate, glutathione, choline, and glycine. Glutamate, a major excitatory neurotransmitter in the mammalian brain, plays a crucial role in memory, learning, cognition, and motor behavior. It also serves as the immediate precursor to gamma-aminobutyric acid (GABA) and glutathione [69,70]. Recent findings from Conde et al. [71] highlight an association between decreased glutamate levels and the exacerbation of pathological cognitive decline. Additionally, Zheng et al. [72] proposed that the development of diabetes-associated cognitive decline in db/db mice is likely linked to a reduction in energy metabolism and disruptions in glutamate-glutamine shuttling between neurons and astrocytes in the hippocampus. Noteworthy is the observation that the expression of postsynaptic glutamate receptors, including the N-methyl-D-aspartate (NMDA) receptor, is diminished in synaptic densities from the brains of chronic streptozotocin-induced type 1 diabetic rats and other animal models with type 2 diabetes [72]. Furthermore, glutamate and aspartate have been identified as two excitatory neurotransmitters related to cognitive impairment [73]. Altogether, there seems to be an implication of glutamate metabolism in DACD, but the detailed relationship of this relationship remains unclear and needs further investigation. L-Alanine, alongside L-Glutamine, L-Lysine, L-Serine, and L-Threonine, has emerged as a prospective biomarker for DACD. These identified biomarkers play integral roles in glycine, serine, and threonine metabolism, as well as in alanine, aspartate, and glutamate metabolism. Furthermore, they contribute to glyoxylate and dicarboxylate metabolism, processes intricately linked with the tricarboxylic acid (TCA) cycle. The identification of these metabolites provides valuable insights into the metabolic pathways implicated in DACD, offering potential avenues for targeted investigations and therapeutic interventions [74]. 

Through our ROC analysis, we were able to develop four different biomarker panels with classification performances exceeding 90%. While the first panel includes four individual metabolites, ((14 or 15)-methylpalmitate (a17:0 or i17:0), isovalerate (i5:0), linolenamide (18:3), and octadecenedioylcarnitine (C18:1-DC)), and has an AUC = 0.943, the inclusion of metabolite ratios (dihomollinolenoylcarnitine (C20:3n3 or 6)/formiminoglutamate, dacylglycerol (16:1/18:2 [2], 16:0/18:3 [1])/formiminoglutamate, 1-linoleoyl-GPA(18:2)/myristoleamide (14:1), and formiminoglutamate/octadecenedioylcarnitine (C18:1DC)) in the other three biomarker panels slightly improved the classification performance to 95%.

We acknowledge the relatively small sample size of patients with MCI and dementia, necessitating their consolidation into a single group. The difference in age between T2D and DACD subjects is also a weakness of this study. These shortcomings limit the generalizability of our results. Lack of information about the medical treatment for both T2D and cognitive decline is also a limitation of this study, as these treatments may influence the metabolomic profiles. Given the small number of MCI and dementia patients, we had to pool them into one group. Future investigations are required to discern and differentiate diabetes-associated metabolic alterations, specifically in MCI and dementia populations, to differentiate mechanisms underlying the onset and progression of disease. It is also important to acknowledge that our metabolite data may be influenced by each patient’s nutritional and metabolic status at the time of this study, given its case–control design.

As previously noted, a limited number of studies have employed peripheral blood to investigate the pathophysiological changes underlying the development of DADC. Comparable to these studies, our investigation involved a modest cohort size. Furthermore, these studies utilized different platforms, biospecimens, and databases for metabolite identification, potentially introducing variations in outcomes. Nonetheless, our findings corroborate certain previously reported observations, particularly alterations in the metabolism of carbohydrates, amino acids, lipids, and sphingolipids. It is noteworthy that our study did not observe significant alterations in uric acid metabolism or bile acid metabolism, which contrasts with the findings reported by [31]. These disparities emphasize the nuanced nature of metabolic changes associated with DADC and underscore the importance of methodological considerations in interpreting and comparing results across studies.

## 4. Material and Methods

### 4.1. Participants

We recruited 54 individuals with T2D and normal cognitive function and 46 individuals with T2D and cognitive decline, which included mild cognitive impairment (MCI) and dementia (Alzheimer’s dementia, vascular dementia, and mixed dementia). This cohort was recruited between 2019 and 2021 as part of a prospective study conducted at the Geriatric and Memory Clinic in Rumailah Hospital (Doha, Qatar).

### 4.2. Plasma Collection

Plasma samples were collected and stored using standardized procedures during the baseline visit. Blood samples were collected in EDTA tubes and centrifuged at 1500× *g* for 15 min at 4 °C with the isolation of plasma, which was stored at −80 °C.

### 4.3. Diagnostic Procedures

All participants were evaluated for memory disorders at a geriatric unit by a team of geriatricians, psychiatrists, and neurologists. The evaluations included clinical assessments, medical history, caregiver interviews, neurological exams, neuropsychological testing, functional evaluations of daily living activities, neuroimaging, and laboratory assessments to rule out other causes of cognitive dysfunction. The diagnosis of MCI and dementia was made according to the International Classification of Diseases, Tenth Revision (ICD-10) criteria. In addition, the diagnosis of Alzheimer’s disease (AD) was based on MRI features typical of AD, with neuroradiologists evaluating hippocampal, entorhinal cortex, and amygdala volume loss based on the Dubois criteria [75]. The diagnosis of vascular dementia (VaD) was based on the National Institute of Neurological Disorders and Stroke-Association Internationale pour la Recherche et l’Enseignement en Neurosciences (NINDS-AIREN) criteria [76], which included the presence of multiple large vessel infarcts or strategically placed single infarcts, multiple lacunes, extensive periventricular white matter lesions, or a combination thereof. The diagnosis of mixed dementia was made when features of AD and significant vascular changes were present.

### 4.4. Cognitive Function Assessment

Cognitive function was assessed using the Montreal Cognitive Assessment (MoCA) test. The MoCA assesses seven cognitive domains, namely visuospatial/executive, naming, memory, attention, language, abstraction, and delayed recall, giving a total score of 30. A score of ≤26 indicates cognitive impairment. A point was added for individuals who had formal education ≤6th grade. Cognitive symptom duration was estimated from the clinical history obtained from relatives and participants.

### 4.5. Functional Independence Assessment

The Functional Independence Measure (FIM) is an 18-point screening test, of which 13 points are for motor and 5 are for cognitive function, and each point is scored from 1 to 7. It was administered by an occupational therapist. The total FIM score ranges from 18 to 126. There is no cutoff point for the FIM, but a higher score indicates greater independence [77].

### 4.6. Metabolomic Profiling

MS-based metabolomic profiling was conducted on the Metabolon platform in Doha, Qatar. The samples were processed and analyzed as previously described [78]. This integrated platform enables high-throughput collection and relative quantitative analysis of analytical data to identify a large number and broad spectrum of molecules with a high degree of confidence. Missing values for the metabolites were imputed using the observed minimum detection value. Prior to performing data analysis, data were Pareto scaled and log transformed. The details pertaining to the full procedures are shown in the Supplementary Data.

### 4.7. Statistical Analysis

Data analyses were performed using stat 16.1 “https://www.stata.com/ (accessed on 30 October 2023)” and Metaboanalyst 5.0 “https://www.metaboanalyst.ca/ (accessed on 30 October 2023)”. We used descriptive statistics to compare the baseline characteristics of the participants. Continuous variables were expressed as the means ± standard deviations (SDs) and compared using the independent sample *t*-test between the two groups. Categorical variables are expressed as percentages, and the chi-squared test was employed to compare the two groups. The odds of dementia were determined by logistic regression adjusted for age and sex and using the metabolites as independent variables. The predictive value of each metabolite was determined by the area under the curve (AUC) in the receiver operating characteristic curve (ROC) analyses. The cutoff point was selected according to the Youden index (sensitivity + specificity − 1). Statistical significance was set at *p* < 0.05.

### 4.8. Metabolomic Analysis

Metabolomic analysis was performed using Metaboanalyst 5.0, “https://www.metaboanalyst.ca (accessed on 30 October 2023)”. The data were log 2 transformed and Pareto scaled. Multivariate analysis was conducted using principal component analysis (PCA) and orthogonal partial least square discrimination analysis (OPLS-DA). Since OPLS-DA is a supervised model, the accuracy of the OPLS-DA model was verified by tenfold cross-validation, and Q^2^ and R^2^ values, which represent the estimate of the predictive ability of the models, are reported. The top 30 most influential metabolites were obtained by variable importance in the projection (VIP). Metabolites with VIP scores >1 are typically considered significant to separate groups. Differential expression of metabolites was assumed if the fold change was >2 and the FDR was <0.05. The significant metabolites identified by VIP analysis were then used for biochemical pathway analysis. The diagnostic performance of the selected classifiers was evaluated using ROC implemented in Metaboanalyst 5.0. Classifier robustness was estimated by the permutation test (1000 times). The relative importance of each metabolite in the predictive models was evaluated using multivariate logistic regression analysis.

### 4.9. Ethical Approval

This study was conducted in accordance with the Declaration of Helsinki and was approved by the Qatar Biomedical Research Institute (2019-013), Hamad Medical Corporation/Weill Cornell Medicine-Qatar (NPRP12S-0213-190080) Institutional Review Boards, following relevant guidelines and regulations, and all participants provided written informed consent.

## 5. Conclusions

Our findings furnish robust evidence delineating the potential attribution of DACD to perturbations in diverse metabolic pathways. Numerous metabolites, intricately linked with pathways, such as the tricarboxylic acid cycle (TCA), as well as the metabolism of amino acids, fatty acids, and carbohydrates, exhibited differential expression between patients with T2D and those with DACD. Pathway analysis unveiled significant alterations in pathways encompassing arginine and unsaturated fatty acid biosynthesis, as well as the metabolism of the citrate cycle, alanine, aspartate, glutamate, alpha-linolenic acid, and glycerophospholipids. These identified perturbations across various metabolic and signaling pathways suggest potential mechanisms, including oxidative stress, neuroinflammation, compromised neuronal protection and plasticity, hippocampal atrophy, heightened vascular damage, and neurodegeneration, as contributors to DACD. Particularly noteworthy is a specific subset of metabolites distinguishing T2D from DACD, possessing the capacity to traverse the blood–brain barrier. Consequently, the analysis of blood-circulating metabolites stands as a promising avenue for elucidating the underlying pathophysiology of diabetes-associated cognitive dysfunction.

Classifier models were developed, achieving AUC-ROC values exceeding 90%, utilizing individual metabolites or a combination of individual metabolites and metabolite ratios, indicating the high potential of blood metabolites as markers of an early diagnosis of DACD.

Further research is imperative to validate and expand on these findings, with a particular focus on exploring the potential of targeted interventions aimed at modulating the identified metabolic pathways. Such interventions hold promise in mitigating the risk of cognitive decline among individuals with diabetes.

## Figures and Tables

**Table 1:** Clinical and laboratory characteristics in participants with and without DACD.

	Diabetes without DACD (n = 54)	Diabetes with DACD (n = 46)	*p*-Value
**Age**	63 (±5)	72 (±7)	**<0.001**
**Female**	24 (44%)	18 (39%)	0.59
**BMI**	31 (±7)	29 (±7)	0.12
**HbA1c mmol**	63 (±15)	55 (±13)	**0.006**
**HbA1c %**	8 (±1)	7 (±1)	**0.006**
**FIM score**	125 (±2)	113 (±18)	**0.032**
**MoCA score**	29 (±1)	21 (±8)	**<0.001**
**VPT**	15 (±11)	17 (±10)	0.49
**SBP**	131 (±17)	131 (±19)	0.90
**DBP**	70 (±10)	69 (±10)	0.50
**TYG**	2 (±1)	2 (±1)	0.32
**HDL**	1 (±0)	1 (±0)	0.066
**LDL**	2 (±1)	2 (±1)	0.85
**TC**	4 (±1)	4 (±1)	0.22
**Creatinine**	75 (±22)	91 (±33)	**0.004**
**Vit D**	26 (±13)	28 (±12)	0.43
**TSH**	2 (±1)	2 (±1)	0.28
**FT4**	14 (±2)	14 (±3)	0.080
**B_12_**	316 (±113)	307 (±129)	0.73
**Hgb**	14 (±2)	13 (±2)	0.52
**MCV**	82 (±6)	86 (±7)	**0.003**

Values are presented as the range or mean ± SD. DACD: diabetes-associated cognitive dysfunction; BMI: body mass index; HDLs: high-density lipoproteins; LDLs: low-density lipoproteins; TC: total cholesterol; TGs: triglycerides; SBP: systolic blood pressure; DBP: diastolic blood pressure. MoCA: Montreal Cognitive Assessment; FIM: Functional Independence Measure. Significant *p*-values at the 5% level are highlighted in bold.

## Data Availability

The data presented in this study are available on request from the corresponding author. The data are not publicly available due to regulatory requirements regarding public disclosure from the providing institute.

## Supplementary Materials

The following supporting information can be downloaded at https://www.mdpi.com/article/10.3390/ijms25042247/s1.

## References

[1] Clasen F., Nunes P.M., Bidkhori G., Bah N., Boeing S., Shoaie S., Anastasiou D. (2023). Systematic diet composition swap in a mouse genome-scale metabolic model reveals determinants of obesogenic diet metabolism in liver cancer. iScience.

[2] Gottesman R.F., Albert M.S., Alonso A., Coker L.H., Coresh J., Davis S.M., Deal J.A., McKhann G.M., Mosley T.H., Sharrett A.R. (2017). Associations Between Midlife Vascular Risk Factors and 25-Year Incident Dementia in the Atherosclerosis Risk in Communities (ARIC) Cohort. JAMA Neurol..

[3] Xue M., Xu W., Ou Y.N., Cao X.P., Tan M.S., Tan L., Yu J.T. (2019). Diabetes mellitus and risks of cognitive impairment and dementia: A systematic review and meta-analysis of 144 prospective studies. Ageing Res. Rev..

[4] Cheng G., Huang C., Deng H., Wang H. (2012). Diabetes as a risk factor for dementia and mild cognitive impairment: A meta-analysis of longitudinal studies. Intern. Med. J..

[5] Li X.Y., Zhang M., Xu W., Li J.Q., Cao X.P., Yu J.T., Tan L. (2019). Midlife Modifiable Risk Factors for Dementia: A Systematic Review and Meta-analysis of 34 Prospective Cohort Studies. Curr. Alzheimer Res..

[6] Pal K., Mukadam N., Petersen I., Cooper C. (2018). Mild cognitive impairment and progression to dementia in people with diabetes, prediabetes and metabolic syndrome: A systematic review and meta-analysis. Soc. Psychiatry Psychiatr. Epidemiol..

[7] Tuligenga R.H., Dugravot A., Tabák A.G., Elbaz A., Brunner E.J., Kivimäki M., Singh-Manoux A. (2014). Midlife type 2 diabetes and poor glycaemic control as risk factors for cognitive decline in early old age: A post-hoc analysis of the Whitehall II cohort study. Lancet Diabetes Endocrinol..

[8] Barbiellini Amidei C., Fayosse A., Dumurgier J., Machado-Fragua M.D., Tabak A.G., van Sloten T., Kivimäki M., Dugravot A., Sabia S., Singh-Manoux A. (2021). Association between Age at Diabetes Onset and Subsequent Risk of Dementia. JAMA.

[9] Haroon N.N., Austin P.C., Shah B.R., Wu J., Gill S.S., Booth G.L. (2015). Risk of dementia in seniors with newly diagnosed diabetes: A population-based study. Diabetes Care.

[10] Crane P.K., Walker R., Hubbard R.A., Li G., Nathan D.M., Zheng H., Haneuse S., Craft S., Montine T.J., Kahn S.E. (2013). Glucose levels and risk of dementia. N. Engl. J. Med..

[11] Ehtewish H., Arredouani A., El-Agnaf O. (2022). Diagnostic, Prognostic, and Mechanistic Biomarkers of Diabetes Mellitus-Associated Cognitive Decline. Int. J. Mol. Sci..

[12] Zhang J.B., Geng N., Li Z.G., Qiao H.J., Sun H.R., Li F. (2016). Biomarkers of Renal Function in Type 2 Diabetic Patients with Cognitive Impairment. Neurosci. Lett..

[13] Huang X., Wang C., Tian S., Huang R., Guo D., Zhang H., Shi J., Wang S. (2019). Higher Plasma Level of Nampt Presaging Memory Dysfunction in Chinese Type 2 Diabetes Patients with Mild Cognitive Impairment. J. Alzheimer’s Dis..

[14] Guo D., Yuan Y., Huang R., Tian S., Wang J., Lin H., An K., Han J., Wang S. (2019). Association between plasma adipsin level and mild cognitive impairment in Chinese patients with type 2 diabetes: A cross-sectional study. BMC Endocr. Disord..

[15] Wang J., Yuan Y., Cai R., Huang R., Tian S., Lin H., Guo D., Wang S. (2018). Association between Plasma Levels of PAI-1, tPA/PAI-1 Molar Ratio, and Mild Cognitive Impairment in Chinese Patients with Type 2 Diabetes Mellitus. J. Alzheimer’s Dis..

[16] Zhu W., Xu L., Zhang H., Tian S., An K., Cao W., Shi J., Tang W., Wang S. (2021). Elevated Plasma Free Fatty Acid Susceptible to Early Cognitive Impairment in Type 2 Diabetes Mellitus. J. Alzheimer’s Dis..

[17] Marioni R.E., Strachan M.W., Reynolds R.M., Lowe G.D., Mitchell R.J., Fowkes F.G., Frier B.M., Lee A.J., Butcher I., Rumley A. (2010). Association between raised inflammatory markers and cognitive decline in elderly people with type 2 diabetes: The Edinburgh Type 2 Diabetes Study. Diabetes.

[18] Gorska-Ciebiada M., Saryusz-Wolska M., Borkowska A., Ciebiada M., Loba J. (2015). Serum levels of inflammatory markers in depressed elderly patients with diabetes and mild cognitive impairment. PLoS ONE.

[19] Gorska-Ciebiada M., Saryusz-Wolska M., Borkowska A., Ciebiada M., Loba J. (2015). C-Reactive Protein, Advanced Glycation End Products, and Their Receptor in Type 2 Diabetic, Elderly Patients with Mild Cognitive Impairment. Front. Aging Neurosci..

[20] Gorska-Ciebiada M., Saryusz-Wolska M., Borkowska A., Ciebiada M., Loba J. (2016). Adiponectin, Leptin and Il-1 B in Elderly Diabetic Patients with Mild Cognitive Impairment. Metab Brain Dis.

[21] Murray A.M., Barzilay J.I., Lovato J.F., Williamson J.D., Miller M.E., Marcovina S., Launer L.J. (2011). Biomarkers of renal function and cognitive impairment in patients with diabetes. Diabetes Care.

[22] Zhen Y.F., Zhang J., Liu X.Y., Fang H., Tian L.B., Zhou D.H., Kosten T.R., Zhang X.Y. (2013). Low BDNF is associated with cognitive deficits in patients with type 2 diabetes. Psychopharmacology.

[23] Murillo Ortíz B., Ramírez Emiliano J., Ramos-Rodríguez E., Martínez-Garza S., Macías-Cervantes H., Solorio-Meza S., Pereyra-Nobara T.A. (2016). Brain-derived neurotrophic factor plasma levels and premature cognitive impairment/dementia in type 2 diabetes. World J. Diabetes.

[24] Zhang A., Sun H., Yan G., Wang P., Wang X. (2015). Metabolomics for Biomarker Discovery: Moving to the Clinic. Biomed. Res. Int..

[25] Nagana Gowda G.A., Raftery D. (2013). Biomarker Discovery and Translation in Metabolomics. Curr. Metabolomics.

[26] Zhang T., Zheng H., Fan K., Xia N., Li J., Yang C., Gao H., Yang Y. (2020). NMR-based metabolomics characterizes metabolic changes in different brain regions of streptozotocin-induced diabetic mice with cognitive decline. Metab. Brain Dis..

[27] Zhao L., Dong M., Ren M., Li C., Zheng H., Gao H. (2018). Metabolomic Analysis Identifies Lactate as an Important Pathogenic Factor in Diabetes-associated Cognitive Decline Rats. Mol. Cell. Proteom..

[28] Du K., Zhai C., Li X., Gang H., Gao X. (2023). Feature-Based Molecular Networking Facilitates the Comprehensive Identification of Differential Metabolites in Diabetic Cognitive Dysfunction Rats. Metabolites.

[29] Chen R., Zeng Y., Xiao W., Zhang L., Shu Y. (2021). LC-MS-Based Untargeted Metabolomics Reveals Early Biomarkers in STZ-Induced Diabetic Rats with Cognitive Impairment. Front. Endocrinol..

[30] Bi T., Zhang L., Zhan L., Feng R., Zhao T., Ren W., Hang T., Zhou W., Lu X. (2022). Integrated Analyses of Microbiomics and Metabolomics Explore the Effect of Gut Microbiota Transplantation on Diabetes-Associated Cognitive Decline in Zucker Diabetic Fatty Rats. Front. Aging Neurosci..

[31] Zhang L., Li M., Zhan L., Lu X., Liang L., Su B., Sui H., Gao Z., Li Y., Liu Y. (2015). Plasma metabolomic profiling of patients with diabetes-associated cognitive decline. PLoS ONE.

[32] Morris J.K., Piccolo B.D., John C.S., Green Z.D., Thyfault J.P., Adams S.H. (2019). Oxylipin Profiling of Alzheimer’s Disease in Nondiabetic and Type 2 Diabetic Elderly. Metabolites.

[33] Sun L., Diao X., Gang X., Lv Y., Zhao X., Yang S., Gao Y., Wang G. (2020). Risk Factors for Cognitive Impairment in Patients with Type 2 Diabetes. J. Diabetes Res..

[34] Koekkoek P.S., Kappelle L.J., van den Berg E., Rutten G.E., Biessels G.J. (2015). Cognitive function in patients with diabetes mellitus: Guidance for daily care. Lancet Neurol..

[35] Shaik M.A., Chan Q.L., Xu J., Xu X., Hui R.J., Chong S.S., Chen C.L., Dong Y. (2016). Risk Factors of Cognitive Impairment and Brief Cognitive Tests to Predict Cognitive Performance Determined by a Formal Neuropsychological Evaluation of Primary Health Care Patients. J. Am. Med. Dir. Assoc..

[36] You Y., Liu Z., Chen Y., Xu Y., Qin J., Guo S., Huang J., Tao J. (2021). The prevalence of mild cognitive impairment in type 2 diabetes mellitus patients: A systematic review and meta-analysis. Acta Diabetol..

[37] Saldana S.L., Guarnaccia C.A. (2022). Comparing cognitive function in white Mexican & non-Hispanic white Americans with/without diabetes. J. Diabetes Metab. Disord..

[38] Zhang X., Jiang X., Han S., Liu Q., Zhou J. (2019). Type 2 Diabetes Mellitus Is Associated with the Risk of Cognitive Impairment: A Meta-Analysis. J. Mol. Neurosci..

[39] Zhang J., Chen C., Hua S., Liao H., Wang M., Xiong Y., Cao F. (2017). An updated meta-analysis of cohort studies: Diabetes and risk of Alzheimer’s disease. Diabetes Res. Clin. Pract..

[40] Hassing L.B., Grant M.D., Hofer S.M., Pedersen N.L., Nilsson S.E., Berg S., McClearn G., Johansson B. (2004). Type 2 diabetes mellitus contributes to cognitive decline in old age: A longitudinal population-based study. J. Int. Neuropsychol. Soc..

[41] Maher P.A., Schubert D.R. (2009). Metabolic links between diabetes and Alzheimer’s disease. Expert Rev. Neurother..

[42] Areosa Sastre A., Vernooij R.W., González-Colaço Harmand M., Martínez G. (2017). Effect of the treatment of Type 2 diabetes mellitus on the development of cognitive impairment and dementia. Cochrane Database Syst. Rev..

[43] Exalto L.G., Biessels G.J., Karter A.J., Huang E.S., Katon W.J., Minkoff J.R., Whitmer R.A. (2013). Risk score for prediction of 10 year dementia risk in individuals with type 2 diabetes: A cohort study. Lancet Diabetes Endocrinol..

[44] Feinkohl I., Price J.F., Strachan M.W., Frier B.M. (2015). The impact of diabetes on cognitive decline: Potential vascular, metabolic, and psychosocial risk factors. Alzheimer’s Res. Ther..

[45] Geijselaers S.L.C., Sep S.J.S., Stehouwer C.D.A., Biessels G.J. (2015). Glucose regulation, cognition, and brain MRI in type 2 diabetes: A systematic review. Lancet Diabetes Endocrinol..

[46] Sredy J., Sawicki D.R., Notvest R.R. (1991). Polyol pathway activity in nervous tissues of diabetic and galactose-fed rats: Effect of dietary galactose withdrawal or tolrestat intervention therapy. J. Diabet. Complicat..

[47] Bril V., Ono Y., Buchanan R.A. (2004). Sural nerve sorbitol in patients with diabetic sensorimotor polyneuropathy. Diabetes Care.

[48] Xu J., Begley P., Church S.J., Patassini S., McHarg S., Kureishy N., Hollywood K.A., Waldvogel H.J., Liu H., Zhang S. (2016). Elevation of brain glucose and polyol-pathway intermediates with accompanying brain-copper deficiency in patients with Alzheimer’s disease: Metabolic basis for dementia. Sci. Rep..

[49] Spagnuolo M.S., Iossa S., Cigliano L. (2020). Sweet but Bitter: Focus on Fructose Impact on Brain Function in Rodent Models. Nutrients.

[50] Johnson R.J., Gomez-Pinilla F., Nagel M., Nakagawa T., Rodriguez-Iturbe B., Sanchez-Lozada L.G., Tolan D.R., Lanaspa M.A. (2020). Cerebral Fructose Metabolism as a Potential Mechanism Driving Alzheimer’s Disease. Front. Aging Neurosci..

[51] Guo L., Chen S., Ou L., Li S., Ye Z.N., Liu H.F. (2022). Disrupted Alpha-Ketoglutarate Homeostasis: Understanding Kidney Diseases from the View of Metabolism and Beyond. Diabetes Metab. Syndr. Obes..

[52] Kostiuchenko O., Lushnikova I., Kowalczyk M., Skibo G. (2022). mTOR/α-ketoglutarate-mediated signaling pathways in the context of brain neurodegeneration and neuroprotection. BBA Adv..

[53] Oldendorf W.H. (1973). Carrier-mediated blood-brain barrier transport of short-chain monocarboxylic organic acids. Am. J. Physiol..

[54] Conn A.R., Steele R.D. (1982). Transport of alpha-keto analogues of amino acids across blood-brain barrier in rats. Am. J. Physiol..

[55] Liu S., He L., Yao K. (2018). The Antioxidative Function of Alpha-Ketoglutarate and Its Applications. Biomed. Res. Int..

[56] Suhre K., Shin S.Y., Petersen A.K., Mohney R.P., Meredith D., Wägele B., Altmaier E., Deloukas P., Erdmann J., Grundberg E. (2011). Human metabolic individuality in biomedical and pharmaceutical research. Nature.

[57] McClay J.L., Vunck S.A., Batman A.M., Crowley J.J., Vann R.E., Beardsley P.M., van den Oord E.J. (2015). Neurochemical Metabolomics Reveals Disruption to Sphingolipid Metabolism Following Chronic Haloperidol Administration. J. Neuroimmune Pharmacol..

[58] Yang J., Yan B., Zhao B., Fan Y., He X., Yang L., Ma Q., Zheng J., Wang W., Bai L. (2020). Assessing the Causal Effects of Human Serum Metabolites on 5 Major Psychiatric Disorders. Schizophr. Bull..

[59] Weng W.C., Huang W.Y., Tang H.Y., Cheng M.L., Chen K.H. (2019). The Differences of Serum Metabolites between Patients with Early-Stage Alzheimer’s Disease and Mild Cognitive Impairment. Front. Neurol..

[60] Chu C.S., Hung C.F., Ponnusamy V.K., Chen K.C., Chen N.C. (2022). Higher Serum DHA and Slower Cognitive Decline in Patients with Alzheimer’s Disease: Two-Year Follow-Up. Nutrients.

[61] Melo van Lent D., Egert S., Wolfsgruber S., Kleineidam L., Weinhold L., Wagner-Thelen H., Maier W., Jessen F., Ramirez A., Schmid M. (2021). Eicosapentaenoic Acid Is Associated with Decreased Incidence of Alzheimer’s Dementia in the Oldest Old. Nutrients.

[62] Wei B.Z., Li L., Dong C.W., Tan C.C., Xu W. (2023). The Relationship of Omega-3 Fatty Acids with Dementia and Cognitive Decline: Evidence from Prospective Cohort Studies of Supplementation, Dietary Intake, and Blood Markers. Am. J. Clin. Nutr..

[63] Otsuka R., Tange C., Nishita Y., Kato Y., Imai T., Ando F., Shimokata H. (2014). Serum docosahexaenoic and eicosapentaenoic acid and risk of cognitive decline over 10 years among elderly Japanese. Eur. J. Clin. Nutr..

[64] Pardeshi R., Bolshette N., Gadhave K., Arfeen M., Ahmed S., Jamwal R., Hammock B.D., Lahkar M., Goswami S.K. (2019). Docosahexaenoic Acid Increases the Potency of Soluble Epoxide Hydrolase Inhibitor in Alleviating Streptozotocin-Induced Alzheimer’s Disease-Like Complications of Diabetes. Front. Pharmacol..

[65] Zhang X., Hu W., Wang Y., Wang W., Liao H., Zhang X., Kiburg K.V., Shang X., Bulloch G., Huang Y. (2022). Plasma metabolomic profiles of dementia: A prospective study of 110,655 participants in the UK Biobank. BMC Med..

[66] de Leeuw F.A., Karamujić-Čomić H., Tijms B.M., Peeters C.F.W., Kester M.I., Scheltens P., Ahmad S., Vojinovic D., Adams H.H.H., Hankemeier T. (2021). Circulating metabolites are associated with brain atrophy and white matter hyperintensities. Alzheimer’s Dement..

[67] Zhang L., Shi L., Shen Y., Miao Y., Wei M., Qian N., Liu Y., Min W. (2019). Spectral tracing of deuterium for imaging glucose metabolism. Nat. Biomed. Eng..

[68] Dong M., Ren M., Li C., Zhang X., Yang C., Zhao L., Gao H. (2018). Analysis of Metabolic Alterations Related to Pathogenic Process of Diabetic Encephalopathy Rats. Front. Cell. Neurosci..

[69] Danbolt N.C. (2001). Glutamate uptake. Prog. Neurobiol..

[70] Pal M.M. (2021). Glutamate: The Master Neurotransmitter and Its Implications in Chronic Stress and Mood Disorders. Front. Hum. Neurosci..

[71] Conde R., Oliveira N., Morais E., Amaral A.P., Sousa A., Graça G., Verde I. (2024). NMR analysis seeking for cognitive decline and dementia metabolic markers in plasma from aged individuals. J. Pharm. Biomed. Anal..

[72] Zheng Y., Yang Y., Dong B., Zheng H., Lin X., Du Y., Li X., Zhao L., Gao H. (2016). Metabonomic profiles delineate potential role of glutamate-glutamine cycle in db/db mice with diabetes-associated cognitive decline. Mol. Brain.

[73] Mazzoli R., Pessione E. (2016). The Neuro-Endocrinological Role of Microbial Glutamate and Gaba Signaling. Front Microbiol..

[74] Zhao Y., Yang Y., Wang D., Wang J., Gao W. (2022). Cerebrospinal Fluid Amino Acid Metabolite Signatures of Diabetic Cognitive Dysfunction Based on Targeted Mass Spectrometry. J. Alzheimer’s Dis..

[75] Dubois B., Picard G., Sarazin M. (2009). Early detection of Alzheimer’s disease: New diagnostic criteria. Dialogues Clin. Neurosci..

[76] Román G.C., Tatemichi T.K., Erkinjuntti T., Cummings J.L., Masdeu J.C., Garcia J.H., Amaducci L., Orgogozo J.M., Brun A., Hofman A. (1993). Vascular dementia: Diagnostic criteria for research studies. Report of the NINDS-AIREN International Workshop. Neurology.

[77] Tanaka N., Nakatsuka M., Ishii H., Nakayama R., Hosaka R., Meguro K. (2013). Clinical utility of the functional independence measure for assessment of patients with Alzheimer’s disease and vascular dementia. Psychogeriatrics.

[78] Evans A.M., DeHaven C.D., Barrett T., Mitchell M., Milgram E. (2009). Integrated, nontargeted ultrahigh performance liquid chromatography/electrospray ionization tandem mass spectrometry platform for the identification and relative quantification of the small-molecule complement of biological systems. Anal. Chem..

