# Supplementary material for: Untargeted Metabolomic Profiling Reveals Differentially Expressed Serum Metabolites and Pathways in Type 2 Diabetes Patients with and without Cognitive Decline: A Cross-Sectional Study
Source: Int J Mol Sci. 2024 Feb 13;25(4):2247. doi: 10.3390/ijms25042247 (PMC10889568; doi:10.3390/ijms25042247)
Supplement: Supplementary file 1 [file ijms-25-02247-s001.zip › ijms-2849768-supplementary.pdf]

Supplementary material

Untargeted Metabolomic Profiling Reveals Differentially Expressed Serum Metabolites and Pathways in Type 2 Diabetes Patients With and Without Cognitive Decline: A Cross-Sectional Study

Neyla AL-AKL[1*], Olfa Khalifa[1], Georgios Ponirakis [3*], Aijaz Parray [5], Marwan Ramadan [6], Shafi Khan [6], Mani Chandran [6], Raheem Ayadathil [5], Ahmed Elsotouhy [5 7], Ahmed Own [5 8], Hanadi Al Hamad [6], Julie Decock [2 9], Nehad M Alajez [2 9], Omar Albagha [2], Rayaz A Malik [3], Omar MA El-Agnaf [2 4], Abdelilah Arredouani [1 2#]

METHODS

Metabolomics profiling

Sample Preparation: The automated MicroLab STAR® system from Hamilton Company was employed for the meticulous preparation of samples. To ensure quality control, several recovery standards were introduced before the initiation of the extraction process. Protein precipitation was performed using methanol with vigorous shaking for 2 minutes (Glen Mills GenoGrinder 2000) to eliminate proteins, dissociate small molecules bound to proteins, or released from the precipitated protein matrix. The resulting solution underwent centrifugation, and the resultant extract was fractionated into five parts: two designated for analysis using separate reverse phase (RP)/UPLC-MS/MS methods with positive ion mode electrospray ionization (ESI), one for analysis utilizing RP/UPLC-MS/MS with negative ion mode ESI, one for analysis employing HILIC/UPLC-MS/MS with negative ion mode ESI, and one sample was reserved for backup purposes. Subsequently, a TurboVap® (Zymark) was briefly employed to eliminate the organic solvent from the sample extracts. Following solvent removal, the sample extracts were stored overnight under a nitrogen atmosphere before undergoing preparation for analysis. This systematic procedure ensured the precise and reproducible extraction of metabolites from the samples, providing a robust foundation for subsequent analytical investigations.

QA/QC: Several categories of controls were concurrently analyzed alongside the experimental samples to uphold the reliability of the study. A pooled matrix sample, generated by combining a small volume from each experimental sample, or alternatively, utilizing a pool of well-characterized human plasma, served as a technical replicate consistently applied throughout the entire dataset. Extracted water samples functioned as process blanks, ensuring the discernment of potential contaminants. Additionally, a meticulously selected cocktail of quality control (QC) standards, intentionally chosen to avoid interference with the measurement of endogenous compounds, was introduced into every analyzed sample. This facilitated the monitoring of instrument performance and aided in chromatographic alignment.

To assess instrument variability, the median relative standard deviation (RSD) was calculated for standards added to each sample before injection into the mass spectrometers. Process variability, on the

other hand, was evaluated by determining the median RSD for all endogenous metabolites (i.e., non-instrument standards) present in 100% of the pooled matrix samples. Experimental samples were judiciously randomized across the platform run, with QC samples strategically interspersed among the injections. This meticulous approach not only ensured the robustness and precision of the analytical process but also guaranteed the validity of the experimental results.

Ultrahigh Performance Liquid Chromatography-Tandem Mass Spectroscopy (UPLC-MS/MS): All methods utilized a Waters ACQUITY ultra-performance liquid chromatography (UPLC) and a Thermo Scientific Q-Exactive high resolution/accurate mass spectrometer interfaced with a heated electrospray ionization (HESI-II) source and Orbitrap mass analyzer operated at 35,000 mass resolution. The sample extract was dried then reconstituted in solvents compatible to each of the four methods. Each reconstitution solvent contained a series of standards at fixed concentrations to ensure injection and chromatographic consistency. One aliquot was analyzed using acidic positive ion conditions, chromatographically optimized for more hydrophilic compounds. In this method, the extract was gradient eluted from a C18 column (Waters UPLC BEH C18-2.1x100 mm, 1.7 µm) using water and methanol, containing 0.05% perfluoropentanoic acid (PFPA) and 0.1% formic acid (FA). Another aliquot was also analyzed using acidic positive ion conditions, however it was chromatographically optimized for more hydrophobic compounds. In this method, the extract was gradient eluted from the same afore mentioned C18 column using methanol, acetonitrile, water, 0.05% PFPA and 0.01% FA and was operated at an overall higher organic content. Another aliquot was analyzed using basic negative ion optimized conditions using a separate dedicated C18 column. The basic extracts were gradient eluted from the column using methanol and water, however with 6.5mM Ammonium Bicarbonate at pH 8. The fourth aliquot was analyzed via negative ionization following elution from a HILIC column (Waters UPLC BEH Amide 2.1x150 mm, 1.7 µm) using a gradient consisting of water and acetonitrile with 10mM Ammonium Formate, pH 10.8. The MS analysis alternated between MS and data-dependent MS^n scans using dynamic exclusion. The scan range varied slighted between methods but covered 70-1000 m/z. Raw data files are archived and extracted as described below.

Data Extraction and Compound Identification: Raw data was extracted, peak-identified and QC processed using Metabolon's hardware and software. These systems are built on a web-service platform utilizing Microsoft's .NET technologies, which run on high-performance application servers and fiber-channel storage arrays in clusters to provide active failover and load-balancing. Compounds were identified by comparison to library entries of purified standards or recurrent unknown entities. Metabolon maintains a library based on authenticated standards that contains the retention time/index (RI), mass to charge ratio (*m/z)*, and chromatographic data (including MS/MS spectral data) on all molecules present in the library. Furthermore, biochemical identifications are based on three criteria: retention index within a narrow RI window of the proposed identification, accurate mass match to the library +/- 10 ppm, and the MS/MS forward and reverse scores between the experimental data and authentic standards. The MS/MS scores are based on a comparison of the ions present in the experimental spectrum to the ions present in the library spectrum. While there may be similarities between these molecules based on one of these factors, the use of all three data points can be utilized to distinguish and differentiate biochemicals. More than 3300 commercially available purified standard compounds have been acquired and registered into LIMS for analysis on all platforms for determination of their analytical characteristics. Additional mass spectral entries have been created for structurally unnamed biochemicals, which have been identified by virtue of their recurrent nature (both chromatographic and mass spectral). These compounds have the potential to be identified by future acquisition of a matching purified standard or by classical structural analysis.

Curation: A variety of curation procedures were carried out to ensure that a high quality data set was made available for statistical analysis and data interpretation. The QC and curation processes were designed to ensure accurate and consistent identification of true chemical entities, and to remove those representing system artifacts, mis-assignments, and background noise. Metabolon data analysts use proprietary visualization and interpretation software to confirm the consistency of peak identification among the various samples. Library matches for each compound were checked for each sample and corrected if necessary.

Metabolite Quantification and Data Normalization: Peaks were quantified using area-under-the-curve. For studies spanning multiple days, a data normalization step was performed to correct variation resulting from instrument inter-day tuning differences. Essentially, each compound was corrected in run-day blocks by registering the medians to equal one (1.00) and normalizing each data point proportionately (termed the "block correction"). For studies that did not require more than one day of analysis, no normalization is necessary, other than for purposes of data visualization.

RESULTS

Table S1. Named metabolites identified in serum samples from DM and DACD individuals

Metabolite NAME	SUPER_PATHWAY	SUB_PATHWAY
aspartate	Amino Acid	Alanine and Aspartate Metabolism
alanine		
asparagine		
N-acetylalanine		
N-acetylaspartate (NAA)		
N-acetylasparagine		
N-carbamoylalanine		
hydroxyasparagine**		
creatinine		Creatine Metabolism
guanidinoacetate		
creatine		
S-1-pyrroline-5-carboxylate		Glutamate Metabolism
glutamate		
glutamine		
N-acetylglutamate		
2-pyrrolidinone		
N-acetylglutamine		
N-acetyl-aspartyl-glutamate (NAAG)		
alpha-ketoglutaramate*		
4-hydroxyglutamate		
carboxyethyl-GABA		
beta-citrylglutamate		
cysteinylglycine		Glutathione Metabolism

5-oxoproline		
2-aminobutyrate		
cys-gly, oxidized		
cysteine-glutathione disulfide		
cysteinylglycine disulfide*		
2-hydroxybutyrate/2-hydroxyisobutyrate		
glycine		Glycine, Serine and Threonine Metabolism
serine		
threonine		
betaine		
dimethylglycine		
sarcosine		
N-acetylglycine		
N-acetylthreonine		
N-acetylserine		
4-guanidinobutanoate		Guanidino and Acetamido Metabolism
1-methylguanidine		
guanidinosuccinate		
histidine		Histidine Metabolism
trans-urocanate		
3-methylhistidine		
imidazole lactate		
1-methylhistidine		
4-imidazoleacetate		
1-methyl-4-imidazoleacetate		
N-acetylhistidine		
1-ribosyl-imidazoleacetate*		
N-acetyl-3-methylhistidine*		
formiminoglutamate		
hydantoin-5-propionate		
1-methyl-5-imidazoleacetate		
imidazole propionate		
N-acetylcarnosine		
N-acetyl-1-methylhistidine*		
N-acetylhistamine		
1-methyl-5-imidazolelactate		
3-hydroxyisobutyrate		Leucine, Isoleucine and Valine Metabolism
isoleucine		
leucine		
valine		
N-acetylleucine		
N-acetylvaline		
beta-hydroxyisovalerate		

methylsuccinate		
ethylmalonate		
3-methyl-2-oxovalerate		
4-methyl-2-oxopentanoate		
alpha-hydroxyisocaproate		
isovalerate (i5:0)		
3-methyl-2-oxobutyrate		
isobutyrylcarnitine (C4)		
3-hydroxy-2-ethylpropionate		
N-acetylisoleucine		
alpha-hydroxyisovalerate		
isovalerylcarnitine (C5)		
isovalerylglycine		
2-methylbutyrylcarnitine (C5)		
2-hydroxy-3-methylvalerate		
isobutyrylglycine		
tiglylcarnitine (C5:1-DC)		
3-methylglutaconate		
3-methylglutarylcarnitine (2)		
methylsuccinoylcarnitine		
2,3-dihydroxy-2-methylbutyrate		
N-carbamoylvaline		
2-ketocaprylate		
N6,N6,N6-trimethyllysine		Lysine Metabolism
2-aminoadipate		
lysine		
pipecolate		
5-hydroxylysine		
N2-acetyllysine		
N6-acetyllysine		
N-acetyl-cadaverine		
5-(galactosylhydroxy)-lysine		
N6-methyllysine		
6-oxopiperidine-2-carboxylate		
fructosyllysine		
N,N,N-trimethyl-5-aminovalerate		
N6,N6-dimethyllysine		
N2-acetyl,N6,N6-dimethyllysine		
N2-acetyl,N6-methyllysine		
N,N-dimethyl-5-aminovalerate		
hydroxy-N6,N6,N6-trimethyllysine*		
N-acetyl-2-aminoadipate		
N-formylmethionine		

S-adenosylhomocysteine (SAH)		Methionine, Cysteine, SAM and Taurine Metabolism
cystine		
cystathionine		
hypotaurine		
3-sulfo-alanine		
methionine		
taurine		
cysteine		
N-acetylmethionine		
methionine sulfoxide		
cysteine s-sulfate		
5-methylthioribose**		
cysteine sulfinic acid		
S-methylmethionine		
S-methylcysteine		
S-methylcysteine sulfoxide		
lanthionine		
N-methyltaurine		
methionine sulfone		
N-acetyltaurine		
2,3-dihydroxy-5-methylthio-4-pentenoate (DMTPA)*		
succinoyltaurine		
2-hydroxy-4-(methylthio)butanoic acid		
S-carboxyethylcysteine		
4-hydroxyphenylacetate		Phenylalanine Metabolism
2-hydroxyphenylacetate		
phenylpyruvate		
phenylalanine		
phenylacetate		
phenyllactate (PLA)		
N-acetylphenylalanine		
N-succinyl-phenylalanine		
spermidine		Polyamine Metabolism
N-acetylputrescine		
5-methylthioadenosine (MTA)		
4-acetamidobutanoate		
acisoga		
(N(1) + N(8))-acetylspermidine		
N-acetyl-isoputreanine		
5-hydroxyindoleacetate		Tryptophan Metabolism
kynurenate		
serotonin		
tryptophan		

picolinate		
anthranilate		
xanthurenate		
kynurenine		
indolelactate		
3-indoxyl sulfate		
indoleacetate		
indolepropionate		
N-acetyltryptophan		
indoleacetylglutamine		
tryptophan betaine		
indole-3-carboxylate		
N-formylanthranilic acid		
N-acetylkynurenine (2)		
C-glycosyltryptophan		
6-bromotryptophan		
oxindolylalanine		
homovanillate (HVA)		Tyrosine Metabolism
3-(4-hydroxyphenyl)lactate		
tyrosine		
thyroxine		
vanillylmandelate (VMA)		
4-hydroxyphenylpyruvate		
3-methoxytyrosine		
gentisate		
N-acetyltyrosine		
phenol sulfate		
3-methoxytyramine sulfate		
N-formylphenylalanine		
tyramine O-sulfate		
vanillic alcohol sulfate		
vanillactate		
p-cresol glucuronide*		
4-methoxyphenol sulfate		
dopamine 4-sulfate		
dopamine 3-O-sulfate		
phenol glucuronide		
arginine		Urea cycle; Arginine and Proline Metabolism
argininosuccinate		
citrulline		
ornithine		
proline		
urea		
trans-4-hydroxyproline		

homoarginine		
homocitrulline		
pro-hydroxy-pro		
N-acetylarginine		
N-acetylproline		
N-acetylcitrulline		
dimethylarginine (SDMA + ADMA)		
N-methylproline		
argininate*		
2-oxoarginine*		
N-delta-acetylornithine		
N2,N5-diacetylornithine		
N-methylhydroxyproline**		
N,N,N-trimethyl-alanylproline betaine (TMAP)		
3-amino-2-piperidone		
N6-carboxymethyllysine	Carbohydrate	Advanced Glycation End-product
N-acetylneuraminate		Aminosugar Metabolism
N-acetylglucosaminylasparagine		
glucuronate		
erythronate*		
N-acetylglucosamine/N-acetylgalactosamine		
sucrose		Disaccharides and Oligosaccharides
mannose		Fructose, Mannose and Galactose Metabolism
fructose		
galactonate		
mannitol/sorbitol		
maltose		Glycogen Metabolism
lactate		Glycolysis, Gluconeogenesis, and Pyruvate Metabolism
glucose		
pyruvate		
glycerate		
1,5-anhydroglucitol (1,5-AG)		
ribitol		Pentose Metabolism
ribonate		
arabonate/xylonate		
arabitol/xylitol		
lyxonate		
ascorbate (vitamin C)	Cofactors and Vitamins	Ascorbate and Aldarate Metabolism
threonate		
gulonate*		
2-O-methylascorbic acid		
ascorbic acid 2-sulfate		

ascorbic acid 3-sulfate*		
biliverdin		Hemoglobin and Porphyrin Metabolism
bilirubin (Z,Z)		
I-urobilinogen		
heme		
bilirubin (E,E)*		
bilirubin (E,Z or Z,E)*		
L-urobilin		
1-methylnicotinamide		Nicotinate and Nicotinamide Metabolism
quinolinate		
nicotinamide		
trigonelline (N'-methylnicotinate)		
N1-methyl-2-pyridone-5-carboxamide		
N1-methyl-4-pyridone-3-carboxamide		
pantothenate		Pantothenate and CoA Metabolism
pantoate		
alpha-tocopherol		Tocopherol Metabolism
gamma-CEHC		
gamma-tocopherol/beta-tocopherol		
retinol (vitamin A)		Vitamin A Metabolism
beta-cryptoxanthin		
carotene diol (1)		
carotene diol (2)		
carotene diol (3)		
pyridoxine (vitamin B6)		Vitamin B6 Metabolism
pyridoxate		
alpha-ketoglutarate	Energy	TCA Cycle
succinate		
fumarate		
malate		
citrate		
aconitate [cis or trans]		
succinylcarnitine (C4-DC)		
citraconate/glutaconate		
2-methylcitrate/homocitrate		
phosphate		Oxidative Phosphorylation
dehydroepiandrosterone sulfate (DHEA-S)	Lipid	Androgenic Steroids
androsterone sulfate		
epiandrosterone sulfate		
5alpha-androstan-3beta,17beta-diol disulfate		

androstenediol (3beta,17beta) disulfate (1)		
androstenediol (3beta,17beta) disulfate (2)		
5alpha-androstan-3alpha,17alpha-diol monosulfate		
5alpha-androstan-3alpha,17beta-diol disulfate		
5alpha-androstan-3alpha,17beta-diol monosulfate (1)		
5alpha-androstan-3beta,17alpha-diol disulfate		
5alpha-androstan-3beta,17beta-diol monosulfate (2)		
androstenediol (3alpha, 17alpha) monosulfate (2)		
androstenediol (3alpha, 17alpha) monosulfate (3)		
androstenediol (3beta,17beta) monosulfate (1)		
androstenediol (3beta,17beta) monosulfate (2)		
16alpha-hydroxy DHEA 3-sulfate		
andro steroid monosulfate C19H28O6S (1)*		
androsterone glucuronide		
5alpha-androstan-3alpha,17beta-diol 17-glucuronide		
etiocholanolone glucuronide		
5alpha-androstan-3alpha,17beta-diol monosulfate (2)		
11beta-hydroxyandrosterone glucuronide		
11beta-hydroxyetiocholanolone glucuronide*		
carnitine		Carnitine Metabolism
deoxycarnitine		
palmitoyl-sphingosine-phosphoethanolamine (d18:1/16:0)		Ceramide PEs
N-palmitoyl-sphingosine (d18:1/16:0)		Ceramides
N-stearoyl-sphingosine (d18:1/18:0)*		
N-palmitoyl-sphingadienine (d18:2/16:0)*		

N-stearoyl-sphingadienine (d18:2/18:0)*		
N-palmitoyl-heptadecasphingosine (d17:1/16:0)*		
ceramide (d18:1/14:0, d16:1/16:0)*		
ceramide (d18:1/17:0, d17:1/18:0)*		
corticosterone		Corticosteroids
cortisone		
cortisol		
cortolone glucuronide (1)		
tetrahydrocortisol glucuronide		
tetrahydrocortisone glucuronide (5)		
oleoyl-linoleoyl-glycerol (18:1/18:2) [1]		Diacylglycerol
oleoyl-linoleoyl-glycerol (18:1/18:2) [2]		
palmitoyl-linoleoyl-glycerol (16:0/18:2) [1]*		
palmitoyl-linoleoyl-glycerol (16:0/18:2) [2]*		
oleoyl-oleoyl-glycerol (18:1/18:1) [2]*		
linoleoyl-arachidonoyl-glycerol (18:2/20:4) [1]*		
linoleoyl-arachidonoyl-glycerol (18:2/20:4) [2]*		
oleoyl-arachidonoyl-glycerol (18:1/20:4) [1]*		
oleoyl-arachidonoyl-glycerol (18:1/20:4) [2]*		
diacylglycerol (16:1/18:2 [2], 16:0/18:3 [1])*		
linoleoyl-linoleoyl-glycerol (18:2/18:2) [1]*		
linoleoyl-linoleoyl-glycerol (18:2/18:2) [2]*		
stearoyl-arachidonoyl-glycerol (18:0/20:4) [1]*		
N-stearoyl-sphinganine (d18:0/18:0)*		Dihydroceramides
N-palmitoyl-sphinganine (d18:0/16:0)		
palmitoyl dihydrosphingomyelin (d18:0/16:0)*		Dihydrosphingomyelins
sphingomyelin (d18:0/18:0, d19:0/17:0)*		

myristoyl dihydrosphingomyelin (d18:0/14:0)*		
sphingomyelin (d18:0/20:0, d16:0/22:0)*		
4-HDoHE		Docosanoid
12-HHTrE		Eicosanoid
12-HETE		
oleoyl ethanolamide		Endocannabinoid
arachidonoyl ethanolamide		
palmitoyl ethanolamide		
N-oleoyltaurine		
N-stearoyltaurine		
N-linoleoyltaurine*		
linoleoyl ethanolamide		
N-palmitoylserine		
N-oleoylserine		
N-stearoylserine*		
octadecenedioylcarnitine (C18:1-DC)*		Fatty Acid Metabolism (Acyl Carnitine, Dicarboxylate)
palmitoylcarnitine (C16)		
cerotoylcarnitine (C26)*		
hexanoylcarnitine (C6)		
octanoylcarnitine (C8)		
decanoylcarnitine (C10)		
laurylcarnitine (C12)		
cis-3,4-methyleneheptanoylcarnitine		
oleoylcarnitine (C18:1)		
myristoleoylcarnitine (C14:1)*		
palmitoleoylcarnitine (C16:1)*		
ximenoylcarnitine (C26:1)*		
5-dodecenoylcarnitine (C12:1)		
linoleoylcarnitine (C18:2)*		
dihomo-linolenoylcarnitine (C20:3n3 or 6)*		
docosahexaenoylcarnitine (C22:6)*		
palmitoylcholine		Fatty Acid Metabolism (Acyl Choline)
oleoylcholine		
arachidonoylcholine		
docosahexaenoylcholine		
palmitoloelycholine		
dihomo-linolenoyl-choline		
eicosapentaenoylcholine		
stearoylcholine*		
linoleoylcholine*		

hexanoylglutamine		Fatty Acid Metabolism (Acyl Glutamine)
4-methylhexanoylglutamine		
hexanoylglycine		Fatty Acid Metabolism (Acyl Glycine)
N-palmitoylglycine		
3-hydroxybutyroylglycine**		
picolinoylglycine		
cis-3,4-methyleneheptanoylglycine		
methylmalonate (MMA)		Fatty Acid Metabolism (also BCAA Metabolism)
propionylglycine		
propionylcarnitine (C3)		
2-methylmalonylcarnitine (C4-DC)		
malonate		Fatty Acid Synthesis
linoleamide (18:2n6)		Fatty Acid, Amide
oleamide		
stearamide (18:0)		
palmitamide (16:0)		
N-linoleoylserine*		
palmitoleamide (16:1)*		
myristamide (14:0)*		
myristoleamide (14:1)*		
margaramide (17:0)*		
heptadecenamide (17:1)*		
linolenamide (18:3)*		
2-aminooctanoate		Fatty Acid, Amino
2-aminoheptanoate		
N-acetyl-2-aminooctanoate*		
isocaproate (i6:0)		Fatty Acid, Branched
(16 or 17)-methylstearate (a19:0 or i19:0)		
(14 or 15)-methylpalmitate (a17:0 or i17:0)		
cis-3,4-methyleneheptanoate		
glutarate (C5-DC)		Fatty Acid, Dicarboxylate
azelate (C9-DC)		
suberate (C8-DC)		
pimelate (C7-DC)		
maleate		
dodecanedioate (C12-DC)		
3-carboxy-4-methyl-5-propyl-2-furanpropanoate (CMPF)		
sebacate (C10-DC)		
3-hydroxydodecanedioate*		
tetradecanedioate (C14-DC)		
hexadecanedioate (C16-DC)		

octadecanedioate (C18-DC)		
3-methyladipate		
2-hydroxyglutarate		
tridecenedioate (C13:1-DC)*		
eicosanedioate (C20-DC)		
docosadioate (C22-DC)		
3-hydroxyadipate		
3-methylglutarate/2-methylglutarate		
hydroxy-CMPF*		
dodecenedioate (C12:1-DC)*		
hexadecenedioate (C16:1-DC)*		
octadecenedioate (C18:1-DC)		
heptenedioate (C7:1-DC)*		
octadecadienedioate (C18:2-DC)*		
3-carboxy-4-methyl-5-pentyl-2-furanpropionate (3-CMPFP)**		
2-hydroxysebacate		
dodecadienoate (12:2)*		
tetradecadienedioate (C14:2-DC)*		
eicosenedioate (C20:1-DC)*		
branched chain 14:0 dicarboxylic acid**		
decadienedioic acid (C10:2-DC)**		
12,13-DiHOME		Fatty Acid, Dihydroxy
9,10-DiHOME		
3,4-dihydroxybutyrate		
2S,3R-dihydroxybutyrate		
2R,3R-dihydroxybutyrate		
2-hydroxystearate		Fatty Acid, Monohydroxy
2-hydroxyoctanoate		
3-hydroxyoctanoate		
8-hydroxyoctanoate		
3-hydroxydecanoate		
3-hydroxylaurate		
3-hydroxysebacate		
5-hydroxyhexanoate		
2-hydroxypalmitate		
alpha-hydroxycaproate		
13-HODE + 9-HODE		
16-hydroxypalmitate		
2-hydroxydecanoate		
3-hydroxyhexanoate		

2-hydroxynervonate*		
2-hydroxyarachidate*		
2-hydroxyheptanoate*		
glycerol		Glycerolipid Metabolism
glycerol 3-phosphate		
glycerophosphoglycerol		
glycosyl-N-stearoyl-sphingosine (d18:1/18:0)		Hexosylceramides (HCER)
glycosyl-N-palmitoyl-sphingosine (d18:1/16:0)		
glycosyl ceramide (d18:2/24:1, d18:1/24:2)*		
glycosyl ceramide (d18:1/20:0, d16:1/22:0)*		
myo-inositol		Inositol Metabolism
chiro-inositol		
3-hydroxybutyrate (BHBA)		Ketone Bodies
acetoacetate		
lactosyl-N-palmitoyl-sphingosine (d18:1/16:0)		Lactosylceramides (LCER)
lactosyl-N-nervonoyl-sphingosine (d18:1/24:1)*		
palmitoleate (16:1n7)		Long Chain Monounsaturated Fatty Acid
erucate (22:1n9)		
myristoleate (14:1n5)		
10-nonadecenoate (19:1n9)		
10-heptadecenoate (17:1n7)		
eicosenoate (20:1)		
oleate/vaccenate (18:1)		
linoleate (18:2n6)		Long Chain Polyunsaturated Fatty Acid (n3 and n6)
arachidonate (20:4n6)		
dihomo-linoleate (20:2n6)		
eicosapentaenoate (EPA; 20:5n3)		
docosahexaenoate (DHA; 22:6n3)		
docosapentaenoate (n3 DPA; 22:5n3)		
docosadienoate (22:2n6)		
adrenate (22:4n6)		
stearidonate (18:4n3)		
linolenate [alpha or gamma; (18:3n3 or 6)]		
docosapentaenoate (n6 DPA; 22:5n6)		
dihomo-linolenate (20:3n3 or n6)		
hexadecadienoate (16:2n6)		

nisinate (24:6n3)		
heneicosapentaenoate (21:5n3)		
tetradecadienoate (14:2)*		
palmitate (16:0)		Long Chain Saturated Fatty Acid
stearate (18:0)		
myristate (14:0)		
margarate (17:0)		
pentadecanoate (15:0)		
1-stearoyl-GPI (18:0)		Lysophospholipid
1-palmitoyl-GPC (16:0)		
1-stearoyl-GPC (18:0)		
1-oleoyl-GPC (18:1)		
1-linoleoyl-GPC (18:2)		
1-palmitoyl-GPA (16:0)		
1-stearoyl-GPE (18:0)		
1-stearoyl-GPG (18:0)		
1-palmitoleoyl-GPC (16:1)*		
1-arachidonoyl-GPC (20:4n6)*		
2-palmitoleoyl-GPC (16:1)*		
2-palmitoyl-GPC (16:0)*		
1-palmitoyl-GPE (16:0)		
1-oleoyl-GPE (18:1)		
1-linoleoyl-GPE (18:2)*		
1-arachidonoyl-GPE (20:4n6)*		
1-arachidonoyl-GPI (20:4)*		
1-palmitoyl-GPI (16:0)		
1-oleoyl-GPI (18:1)		
1-linoleoyl-GPI (18:2)*		
2-stearoyl-GPE (18:0)*		
1-stearoyl-GPS (18:0)*		
1-arachidonoyl-GPA (20:4)		
1-linolenoyl-GPC (18:3)*		
1-palmitoyl-GPG (16:0)*		
1-linoleoyl-GPA (18:2)*		
1-linoleoyl-GPG (18:2)*		
1-(1-enyl-palmitoyl)-GPC (P-16:0)*		Lysoplasmalogen
1-(1-enyl-palmitoyl)-GPE (P-16:0)*		
1-(1-enyl-stearoyl)-GPE (P-18:0)*		
1-(1-enyl-oleoyl)-GPE (P-18:1)*		
laurate (12:0)		Medium Chain Fatty Acid
caprate (10:0)		
heptanoate (7:0)		

caprylate (8:0)		
10-undecenoate (11:1n1)		
5-dodecenoate (12:1n7)		
cis-4-decenoate (10:1n6)*		
(2 or 3)-decenoate (10:1n7 or n8)		
3-hydroxy-3-methylglutarate		Mevalonate Metabolism
1-palmitoylglycerol (16:0)		Monoacylglycerol
1-oleoylglycerol (18:1)		
2-oleoylglycerol (18:1)		
2-linoleoylglycerol (18:2)		
1-linoleoylglycerol (18:2)		
1-arachidonylglycerol (20:4)		
1-linolenoylglycerol (18:3)		
1-docosahexaenoylglycerol (22:6)		
1-myristoylglycerol (14:0)		
1-dihomo-linolenylglycerol (20:3)		
1-palmitoleoylglycerol (16:1)*		
1-palmitoyl-2-linoleoyl-GPC (16:0/18:2)		Phosphatidylcholine (PC)
1-palmitoyl-2-oleoyl-GPC (16:0/18:1)		
1,2-dipalmitoyl-GPC (16:0/16:0)		
1-myristoyl-2-palmitoyl-GPC (14:0/16:0)		
1-stearoyl-2-arachidonoyl-GPC (18:0/20:4)		
1,2-dilinoleoyl-GPC (18:2/18:2)		
1-stearoyl-2-oleoyl-GPC (18:0/18:1)		
1-palmitoyl-2-arachidonoyl-GPC (16:0/20:4n6)		
1-palmitoyl-2-docosahexaenoyl-GPC (16:0/22:6)		
1-stearoyl-2-docosahexaenoyl-GPC (18:0/22:6)		
1-palmitoyl-2-stearoyl-GPC (16:0/18:0)		
1-stearoyl-2-linoleoyl-GPC (18:0/18:2)*		
1-palmitoyl-2-palmitoleoyl-GPC (16:0/16:1)*		
1-palmitoyl-2-dihomo-linolenoyl-GPC (16:0/20:3n3 or 6)*		
1-oleoyl-2-docosahexaenoyl-GPC (18:1/22:6)*		

1-linoleoyl-2-arachidonoyl-GPC (18:2/20:4n6)*		
1-myristoyl-2-arachidonoyl-GPC (14:0/20:4)*		
1-linoleoyl-2-linolenoyl-GPC (18:2/18:3)*		
1-palmitoyl-2-oleoyl-GPE (16:0/18:1)		Phosphatidylethanolamine (PE)
1-stearoyl-2-oleoyl-GPE (18:0/18:1)		
1-palmitoyl-2-linoleoyl-GPE (16:0/18:2)		
1-stearoyl-2-linoleoyl-GPE (18:0/18:2)*		
1-stearoyl-2-arachidonoyl-GPE (18:0/20:4)		
1-palmitoyl-2-arachidonoyl-GPE (16:0/20:4)*		
1-palmitoyl-2-docosahexaenoyl-GPE (16:0/22:6)*		
1-stearoyl-2-docosahexaenoyl-GPE (18:0/22:6)*		
1-oleoyl-2-linoleoyl-GPE (18:1/18:2)*		
1,2-dilinoleoyl-GPE (18:2/18:2)*		
1-palmitoyl-2-linoleoyl-GPI (16:0/18:2)		Phosphatidylinositol (PI)
1-stearoyl-2-arachidonoyl-GPI (18:0/20:4)		
1-stearoyl-2-linoleoyl-GPI (18:0/18:2)		
1-palmitoyl-2-oleoyl-GPI (16:0/18:1)*		
phosphoethanolamine		Phospholipid Metabolism
choline		
glycerophosphorylcholine (GPC)		
glycerophosphoethanolamine		
glycerophosphoinositol*		
trimethylamine N-oxide		
glycerophosphoserine*		
1-(1-enyl-stearoyl)-2-oleoyl-GPE (P-18:0/18:1)		Plasmalogen
1-(1-enyl-stearoyl)-2-arachidonoyl-GPE (P-18:0/20:4)*		
1-(1-enyl-palmitoyl)-2-arachidonoyl-GPE (P-16:0/20:4)*		
1-(1-enyl-palmitoyl)-2-oleoyl-GPE (P-16:0/18:1)*		

1-(1-enyl-palmitoyl)-2-oleoyl-GPC (P-16:0/18:1)*		
1-(1-enyl-palmitoyl)-2-linoleoyl-GPC (P-16:0/18:2)*		
1-(1-enyl-palmitoyl)-2-arachidonoyl-GPC (P-16:0/20:4)*		
1-(1-enyl-palmitoyl)-2-linoleoyl-GPE (P-16:0/18:2)*		
1-(1-enyl-palmitoyl)-2-palmitoleoyl-GPC (P-16:0/16:1)*		
1-(1-enyl-palmitoyl)-2-palmitoyl-GPC (P-16:0/16:0)*		
1-(1-enyl-stearoyl)-2-linoleoyl-GPE (P-18:0/18:2)*		
pregnenediol disulfate (C21H34O8S2)*		Pregnenolone Steroids
21-hydroxypregnenolone disulfate		
pregnenediol sulfate (C21H34O5S)*		
17alpha-hydroxypregnenolone 3-sulfate		
pregnenolone sulfate		
17alpha-hydroxypregnanolone glucuronide		
pregnenetriol sulfate*		
pregnenetriol disulfate*		
cholate		Primary Bile Acid Metabolism
glycocholate		
glycochenodeoxycholate		
taurochenodeoxycholate		
taurocholate		
tauro-beta-muricholate		
glycochenodeoxycholate 3-sulfate		
glycocholate glucuronide (1)		
glycochenodeoxycholate glucuronide (1)		
glyco-beta-muricholate**		
cholic acid glucuronide		
5alpha-pregnan-3beta,20alpha-diol disulfate		Progestin Steroids
5alpha-pregnan-3beta,20beta-diol monosulfate (1)		
5alpha-pregnan-3beta,20alpha-diol monosulfate (2)		
5alpha-pregnan-3beta-ol,20-one sulfate		

pregnanediol-3-glucuronide		
pregnanolone/allopregnanolone sulfate		
deoxycholate		Secondary Bile Acid Metabolism
ursodeoxycholate		
taurodeoxycholate		
glycodeoxycholate		
glycolithocholate		
hyocholate		
glycolithocholate sulfate*		
taurolithocholate 3-sulfate		
glycocholenate sulfate*		
taurocholenate sulfate*		
isoursodeoxycholate		
glycoursodeoxycholate		
tauroursodeoxycholate		
glycohyocholate		
3beta-hydroxy-5-cholenoate		
glycodeoxycholate 3-sulfate		
deoxycholic acid glucuronide		
lithocholate sulfate (1)		
taurochenodeoxycholic acid 3-sulfate		
deoxycholic acid 12-sulfate*		
sphinganine		Sphingolipid Synthesis
sphinganine-1-phosphate		
sphingadienine		
stearoyl sphingomyelin (d18:1/18:0)		Sphingomyelins
sphingomyelin (d18:1/18:1, d18:2/18:0)		
palmitoyl sphingomyelin (d18:1/16:0)		
sphingomyelin (d18:1/14:0, d16:1/16:0)*		
sphingomyelin (d18:2/16:0, d18:1/16:1)*		
sphingomyelin (d18:2/14:0, d18:1/14:1)*		
sphingomyelin (d18:1/24:1, d18:2/24:0)*		
sphingomyelin (d18:1/20:0, d16:1/22:0)*		
sphingomyelin (d18:1/20:1, d18:2/20:0)*		
sphingomyelin (d18:1/20:2, d18:2/20:1, d16:1/22:2)*		

behenoyl sphingomyelin (d18:1/22:0)*		
sphingomyelin (d18:1/22:1, d18:2/22:0, d16:1/24:1)*		
sphingomyelin (d18:1/22:2, d18:2/22:1, d16:1/24:2)*		
lignoceroyl sphingomyelin (d18:1/24:0)		
sphingomyelin (d17:1/16:0, d18:1/15:0, d16:1/17:0)*		
sphingomyelin (d18:1/17:0, d17:1/18:0, d19:1/16:0)		
tricosanoyl sphingomyelin (d18:1/23:0)*		
sphingomyelin (d18:2/23:0, d18:1/23:1, d17:1/24:1)*		
sphingomyelin (d18:2/24:1, d18:1/24:2)*		
sphingomyelin (d18:1/21:0, d17:1/22:0, d16:1/23:0)*		
sphingomyelin (d18:1/19:0, d19:1/18:0)*		
sphingomyelin (d18:2/18:1)*		
sphingomyelin (d18:2/24:2)*		
sphingomyelin (d18:2/21:0, d16:2/23:0)*		
sphingomyelin (d18:2/23:1)*		
sphingomyelin (d17:2/16:0, d18:2/15:0)*		
sphingomyelin (d17:1/14:0, d16:1/15:0)*		
hydroxypalmitoyl sphingomyelin (d18:1/16:0(OH))**		
sphingosine 1-phosphate		Sphingosines
cholesterol		Sterol
beta-sitosterol		
cholesterol sulfate		
7alpha-hydroxy-3-oxo-4-cholestenoate (7-Hoca)		
3beta,7alpha-dihydroxy-5-cholestenoate		
4-cholesten-3-one		
3beta-hydroxy-5-cholestenoate		
methylphosphate	Nucleotide	Purine and Pyrimidine Metabolism
hypoxanthine		Purine Metabolism, (Hypo)Xanthine/Inosine containing
allantoic acid		
inosine		
allantoin		

xanthine		
urate		
N1-methylinosine		
adenosine 5'-monophosphate (AMP)		Purine Metabolism, Adenine containing
N6-methyladenosine		
adenosine		
adenine		
N1-methyladenosine		
N6-carbamoylthreonyladenosine		
guanosine		Purine Metabolism, Guanine containing
N2,N2-dimethylguanosine		
7-methylguanine		
cytidine		Pyrimidine Metabolism, Cytidine containing
cytosine		
3-methylcytidine		
2'-O-methylcytidine		
orotate		Pyrimidine Metabolism, Orotate containing
dihydroorotate		
N-carbamoylaspartate		
orotidine		
5,6-dihydrothymine		Pyrimidine Metabolism, Thymine containing
3-aminoisobutyrate		
beta-alanine		Pyrimidine Metabolism, Uracil containing
uridine		
2'-deoxyuridine		
pseudouridine		
uracil		
3-ureidopropionate		
5,6-dihydrouracil		
5,6-dihydrouridine		
3-(3-amino-3-carboxypropyl)uridine*		
5-methyluridine (ribothymidine)		
N-acetyl-beta-alanine		
2'-O-methyluridine		
glutamine_degradant*	Partially Characterized Molecules	Partially Characterized Molecules
glucuronide of C10H18O2 (1)*		
glucuronide of C10H18O2 (7)*		
glucuronide of C10H18O2 (8)*		
glycine conjugate of C10H14O2 (1)*		
glutamine conjugate of C7H12O2*		

glutamine conjugate of C6H10O2 (1)*		
glutamine conjugate of C6H10O2 (2)*		
metabolonic lactone sulfate		
pentose acid*		
branched-chain, straight-chain, or cyclopropyl 10:1 fatty acid (1)*		
branched-chain, straight-chain, or cyclopropyl 10:1 fatty acid (2)*		
branched-chain, straight-chain, or cyclopropyl 12:1 fatty acid*		
bilirubin degradation product, C16H18N2O5 (1)**		
bilirubin degradation product, C16H18N2O5 (2)**		
bilirubin degradation product, C17H18N2O4 (1)**		
bilirubin degradation product, C17H18N2O4 (2)**		
bilirubin degradation product, C17H18N2O4 (3)**		
bilirubin degradation product, C17H20N2O5 (1)**		
bilirubin degradation product, C17H20N2O5 (2)**		
bilirubin degradation product, C16H18N2O5 (3)**		
bilirubin degradation product, C16H18N2O5 (4)**		
phenylacetylglycine	Peptide	Acetylated Peptides
phenylacetylglutamine		
phenylacetylglutamate		
4-hydroxyphenylacetylglutamine		
glycylvaline		Dipeptide
threonylphenylalanine		
cyclo(leu-pro)		
cyclo(his-pro)		
leucylglycine		
phenylalanylglycine		
prolylglycine		
fibrinopeptide A*		Fibrinogen Cleavage Peptide
fibrinopeptide A, des-ala(1)*		
fibrinopeptide A, phosphono-ser(3)*		
fibrinopeptide A (5-16)*		

fibrinopeptide A (7-16)*		
fibrinopeptide A (3-15)**		
fibrinopeptide A (2-15)**		
fibrinopeptide A (3-16)**		
fibrinopeptide A (4-15)**		
fibrinopeptide A (8-16)**		
fibrinopeptide B (1-13)**		
fibrinopeptide B (1-12)**		
fibrinopeptide B (1-11)**		
fibrinopeptide B (1-9)**		
gamma-glutamylglutamate		Gamma-glutamyl Amino Acid
gamma-glutamyltyrosine		
gamma-glutamylglutamine		
gamma-glutamylhistidine		
gamma-glutamylleucine		
gamma-glutamylphenylalanine		
gamma-glutamylvaline		
gamma-glutamyl-epsilon-lysine		
gamma-glutamylglycine		
gamma-glutamyltryptophan		
gamma-glutamylmethionine		
gamma-glutamylthreonine		
gamma-glutamylisoleucine*		
gamma-glutamyl-2-aminobutyrate		
gamma-glutamyl-alpha-lysine		
gamma-glutamylcitrulline*		
N,N-dimethyl-pro-pro		Modified Peptides
HWESASXX*		Polypeptide
glu-gly-asn-val**		
1H-indole-7-acetic acid	Xenobiotics	Bacterial/Fungal
N-methylpipecolate		
2-hydroxyhippurate (salicylurate)		Benzoate Metabolism
benzoate		
3-phenylpropionate (hydrocinnamate)		
hippurate		
4-acetylphenol sulfate		
p-cresol sulfate		
4-hydroxyhippurate		
catechol sulfate		
3-(3-hydroxyphenyl)propionate		
4-vinylphenol sulfate		
4-ethylphenylsulfate		

o-cresol sulfate			
3-hydroxyhippurate			
3-methyl catechol sulfate (2)			
4-methylcatechol sulfate			
3-methyl catechol sulfate (1)			
guaiacol sulfate			
3-(3-hydroxyphenyl)propionate sulfate			
4-methylguaiacol sulfate			
methyl-4-hydroxybenzoate sulfate			
propyl 4-hydroxybenzoate sulfate			
3-methoxycatechol sulfate (1)			
3-methoxycatechol sulfate (2)			
3-hydroxyhippurate sulfate			
4-ethylcatechol sulfate			
4-allylcatechol sulfate			
4-vinylcatechol sulfate			
2-methoxyhydroquinone sulfate (1)			
2-methoxyhydroquinone sulfate (2)			
triethanolamine		Chemical	
sulfate*			
dimethyl sulfone			
3-acetylphenol sulfate			
meglumine			
O-sulfo-tyrosine			
4-hydroxychlorothalonil			
3-hydroxypyridine sulfate			
2-methoxyresorcinol sulfate			
6-hydroxyindole sulfate			
gentisic acid-5-glucoside			
1,2,3-benzenetriol sulfate (1)			
1,2,3-benzenetriol sulfate (2)			
thioproline			
perfluorooctanesulfonate (PFOS)			
perfluorooctanoate (PFOA)			
2,2'-methylenebis(6-tert-butyl-p-cresol)			
2-naphthol sulfate			
3-hydroxypyridine glucuronide			
5-hydroxy-2-methylpyridine sulfate			

3-hydroxy-2-methylpyridine sulfate		
2-acrylamidoglycolic acid		
3,5-dichloro-2,6-dihydroxybenzoic acid		
3-bromo-5-chloro-2,6-dihydroxybenzoic acid*		
2,4-di-tert-butylphenol		
perfluorohexanesulfonate (PFHxS)		
4-acetamidophenol		Drug - Analgesics, Anesthetics
ibuprofen		
3-(N-acetyl-cystein-S-yl) acetaminophen		
4-acetaminophen sulfate		
4-acetamidophenylglucuronide		
salicyluric glucuronide*		
2-hydroxyacetaminophen sulfate*		
2-methoxyacetaminophen sulfate*		
2-methoxyacetaminophen glucuronide*		
3-(cystein-S-yl)acetaminophen*		
celecoxib		
carboxyibuprofen		
3-(methylthio)acetaminophen sulfate*		
amoxicillin		Drug - Antibiotic
fluconazole		
triclosan sulfate		
clarithromycin		
metoprolol		Drug - Cardiovascular
metoprolol acid metabolite*		
nifedipine		
4-hydroxycoumarin		
atenolol		
hydrochlorothiazide		
warfarin		
furosemide		
chlorthalidone		
valsartan		
alpha-hydroxymetoprolol		
rivaroxaban		
7-hydroxywarfarin		
losartan		
omeprazole		Drug - Gastrointestinal

ranitidine		
pantoprazole		
dexlansoprazole		
ranitidine N-oxide*		
metformin		Drug - Metabolic
oxypurinol		
allopurinol		
sitagliptin		
glyburide		
glipizide		
glimepiride		
gliclazide		
valproate		Drug - Neurological
gabapentin		
pregabalin		
levetiracetam		
3-hydroxyvalproate		
donepezil		
galantamine		
1-hydroxy-2-naphthalenecarboxylate		Drug - Other
mirtazapine		Drug - Psychoactive
5-hydroxy-6-methoxy duloxetine sulfate*		
fexofenadine		Drug - Respiratory
cetirizine		
montelukast		
salicylate		Drug - Topical Agents
hydroquinone sulfate		
2,6-dihydroxybenzoic acid		
gluconate		Food Component/Plant
methyl indole-3-acetate		
tartarate		
2-isopropylmalate		
quinate		
levulinate (4-oxovalerate)		
theanine		
tartronate (hydroxymalonate)		
erythritol		
N-(2-furoyl)glycine		
piperine		
stachydrine		
homostachydrine*		
thymol sulfate		
pyrraline		

4-allylphenol sulfate		
ergothioneine		
cinnamoylglycine		
2,3-dihydroxyisovalerate		
dihydroferulate		
alliin		
mannonate*		
histidine betaine (hercynine)*		
2-piperidinone		
indolin-2-one		
2-aminophenol sulfate		
S-allylcysteine		
N-acetylalliin		
ferulic acid 4-sulfate		
methyl glucopyranoside (alpha + beta)		
isoeugenol sulfate		
4-vinylguaiacol sulfate		
eugenol sulfate		
2-acetamidophenol sulfate		
acesulfame		
umbelliferone sulfate		
caffeic acid sulfate		
3-hydroxystachydrine*		
3-formylindole		
dihydroferulic acid sulfate		
3-indoleglyoxylic acid		
ethyl beta-glucopyranoside		
glucuronide of piperine metabolite C17H21NO3 (4)*		
sulfate of piperine metabolite C16H19NO3 (2)*		
sulfate of piperine metabolite C16H19NO3 (3)*		
sulfate of piperine metabolite C18H21NO3 (1)*		
sulfate of piperine metabolite C18H21NO3 (3)*		
N-acetyltheanine		
(2,4 or 2,5)-dimethylphenol sulfate		
dihydrocaffeate sulfate (2)		
3-ethylcatechol sulfate (1)		
4-acetylcatechol sulfate (1)		
(S)-a-amino-omega-caprolactam		
vanillic acid glycine		

menthol glucuronide		
cotinine		Tobacco Metabolite
hydroxycotinine		
caffeine		Xanthine Metabolism
theophylline		
theobromine		
paraxanthine		
1,3-dimethylurate		
3-methylxanthine		
7-methylxanthine		
1,3,7-trimethylurate		
1,7-dimethylurate		
1-methylurate		
5-acetylamino-6-amino-3-methyluracil		
1-methylxanthine		
7-methylurate		

Table S2. Metabolites with VIP>1 based on OPLSDA

Metabolite name	VIP
palmitoleamide (16:1)*	3.23
(14 or 15)-methylpalmitate (a17:0 or i17:0)	3.18
linolenamide (18:3)*	3.14
myristoleamide (14:1)*	3.10
myristamide (14:0)*	2.93
1-(1-enyl-palmitoyl)-2-palmitoleoyl-GPC (P-16:0/16:1)*	2.75
lignoceroyl sphingomyelin (d18:1/24:0)	2.74
L-urobilin	2.64
dopamine 3-O-sulfate	2.60
sphinganine-1-phosphate	2.60
12-HHTrE	2.60
stearamide (18:0)	2.57
N-stearoyltaurine	2.52
linoleamide (18:2n6)	2.52
formiminoglutamate	2.49

margaramide (17:0)*	2.49
N-oleoyltaurine	2.44
palmitamide (16:0)	2.43
tricosanoyl sphingomyelin (d18:1/23:0)*	2.40
galactonate	2.40
1-linoleoyl-GPA (18:2)*	2.40
2-pyrrolidinone	2.39
isocaproate (i6:0)	2.39
guanidinosuccinate	2.37
I-urobilinogen	2.35
N-palmitoylglycine	2.35
menthol glucuronide	2.31
diacylglycerol (16:1/18:2 [2], 16:0/18:3 [1])*	2.29
isovalerate (i5:0)	2.29
cysteinylglycine	2.29
2-stearoyl-GPE (18:0)*	2.28
gluconate	2.24
1-stearoyl-2-linoleoyl-GPE (18:0/18:2)*	2.23
mannose	2.20
octadecenedioylcarnitine (C18:1-DC)*	2.19
palmitate (16:0)	2.17
N-(2-furoyl)glycine	2.17
5alpha-androstan-3beta,17beta-diol monosulfate (2)	2.15
oxindolylalanine	2.14
glucose	2.14
2,3-dihydroxy-2-methylbutyrate	2.12
fibrinopeptide A*	2.12
gamma-glutamyltryptophan	2.11
palmitoleate (16:1n7)	2.10
mannonate*	2.10
N-palmitoyl-heptadecasphingosine (d17:1/16:0)*	2.07
5alpha-androstan-3alpha,17beta-diol monosulfate (1)	2.05
phenylacetylglycine	2.05
10-heptadecenoate (17:1n7)	2.03
cis-3,4-methyleneheptanoate	2.00
branched-chain, straight-chain, or cyclopropyl 12:1 fatty acid*	1.99
1-oleoyl-GPE (18:1)	1.97
5alpha-pregnan-3beta,20beta-diol monosulfate (1)	1.97

1-arachidonylglycerol (20:4)	1.97
dopamine 4-sulfate	1.95
fumarate	1.94
myristoleate (14:1n5)	1.92
5-hydroxyhexanoate	1.91
alpha-ketoglutarate	1.89
oleate/vaccenate (18:1)	1.89
citrate	1.88
vanillactate	1.87
myristate (14:0)	1.87
3-hydroxy-2-methylpyridine sulfate	1.87
5-dodecenoate (12:1n7)	1.86
glycosyl-N-stearoyl-sphingosine (d18:1/18:0)	1.84
androstenediol (3alpha, 17alpha) monosulfate (2)	1.83
(2 or 3)-decenoate (10:1n7 or n8)	1.82
fibrinopeptide A, des-ala(1)*	1.82
tetradecadienoate (14:2)*	1.82
gulonate*	1.81
bilirubin degradation product, C16H18N2O5 (4)**	1.79
3-methylglutarate/2-methylglutarate	1.79
arachidonoyl ethanolamide	1.78
aconitate [cis or trans]	1.78
laurate (12:0)	1.75
androstenediol (3beta,17beta) monosulfate (2)	1.75
tartronate (hydroxymalonate)	1.74
10-nonadecenoate (19:1n9)	1.73
bilirubin degradation product, C16H18N2O5 (3)**	1.73
dihomo-linolenoylcarnitine (C20:3n3 or 6)*	1.72
linoleate (18:2n6)	1.72
1-linoleoyl-2-linolenoyl-GPC (18:2/18:3)*	1.72
(S)-a-amino-omega-caprolactam	1.71
(16 or 17)-methylstearate (a19:0 or i19:0)	1.71
11beta-hydroxyetiocholanolone glucuronide*	1.71
4-ethylcatechol sulfate	1.71
pimelate (C7-DC)	1.71
succinylcarnitine (C4-DC)	1.71
ximenoylcarnitine (C26:1)*	1.70
androsterone sulfate	1.70
sphingadienine	1.70

2-linoleoylglycerol (18:2)	1.70
4-vinylguaiacol sulfate	1.70
docosapentaenoate (n3 DPA; 22:5n3)	1.69
threonylphenylalanine	1.69
eicosapentaenoate (EPA; 20:5n3)	1.68
5,6-dihydrouridine	1.67
cholesterol sulfate	1.66
N-acetylleucine	1.66
N-acetyltheanine	1.65
stearate (18:0)	1.65
5alpha-androstan-3beta,17alpha-diol disulfate	1.65
cis-4-decenoate (10:1n6)*	1.65
hydantoin-5-propionate	1.64
1-arachidonoyl-GPA (20:4)	1.64
1-palmitoyl-GPA (16:0)	1.64
linoleoyl-arachidonoyl-glycerol (18:2/20:4) [2]*	1.63
hexanoylcarnitine (C6)	1.63
1H-indole-7-acetic acid	1.62
3-methoxytyramine sulfate	1.62
tetradecadienedioate (C14:2-DC)*	1.62
dihomo-linoleate (20:2n6)	1.62
N6-carboxymethyllysine	1.62
mannitol/sorbitol	1.62
fructose	1.61
dodecadienoate (12:2)*	1.61
heme	1.60
N2,N5-diacetylornithine	1.60
N-acetylkynurenine (2)	1.59
tiglylcarnitine (C5:1-DC)	1.58
5alpha-androstan-3alpha,17alpha-diol monosulfate	1.58
stearidonate (18:4n3)	1.57
pentadecanoate (15:0)	1.57
nisinate (24:6n3)	1.57
heptadecenamide (17:1)*	1.55
N6,N6-dimethyllysine	1.55
linolenate [alpha or gamma; (18:3n3 or 6)]	1.55
hexadecadienoate (16:2n6)	1.53
17alpha-hydroxypregnenolone 3-sulfate	1.53
glycocholate glucuronide (1)	1.53

pregnanolone/allopregnanolone sulfate	1.52
eicosenoate (20:1)	1.52
methylsuccinoylcarnitine	1.50
glycine conjugate of C10H14O2 (1)*	1.50
margarate (17:0)	1.50
N-carbamoylvaline	1.49
3-hydroxy-3-methylglutarate	1.48
cytosine	1.48
1-linoleoylglycerol (18:2)	1.48
docosahexaenoate (DHA; 22:6n3)	1.47
pseudouridine	1.46
propyl 4-hydroxybenzoate sulfate	1.46
ferulic acid 4-sulfate	1.46
1,2-dilinoleoyl-GPE (18:2/18:2)*	1.46
glucuronate	1.45
hippurate	1.45
cis-3,4-methyleneheptanoylglycine	1.44
16-hydroxypalmitate	1.44
perfluorooctanesulfonate (PFOS)	1.43
2-methylmalonylcarnitine (C4-DC)	1.43
uridine	1.43
N-stearoyl-sphingosine (d18:1/18:0)*	1.43
N6-carbamoylthreonyladenosine	1.42
alpha-tocopherol	1.41
guaiacol sulfate	1.40
homocitrulline	1.39
N-delta-acetylornithine	1.38
orotate	1.37
1-linoleoyl-GPE (18:2)*	1.37
caffeic acid sulfate	1.37
3-hydroxypyridine sulfate	1.36
propionylcarnitine (C3)	1.36
octanoylcarnitine (C8)	1.36
4-acetylphenol sulfate	1.36
1-palmitoyl-2-oleoyl-GPE (16:0/18:1)	1.36
orotidine	1.36
cinnamoylglycine	1.35
4-HDoHE	1.35
fructosyllysine	1.35

lactate	1.34
salicyluric glucuronide*	1.33
vanillic acid glycine	1.33
ibuprofen	1.32
heneicosapentaenoate (21:5n3)	1.31
2-hydroxyphenylacetate	1.30
dihomo-linolenate (20:3n3 or n6)	1.30
lithocholate sulfate (1)	1.30
methylsuccinate	1.29
hypoxanthine	1.27
quinate	1.27
corticosterone	1.27
phenylacetylglutamine	1.27
N-linoleoyltaurine*	1.27
arachidonoylcholine	1.26
2-hydroxypalmitate	1.26
2R,3R-dihydroxybutyrate	1.26
phenyllactate (PLA)	1.26
palmitoyl ethanolamide	1.25
docosadienoate (22:2n6)	1.25
fibrinopeptide A (3-16)**	1.25
valsartan	1.25
dodecanedioate (C12-DC)	1.24
3-(3-hydroxyphenyl)propionate	1.23
3-hydroxyadipate	1.23
pentose acid*	1.23
indoleacetylglutamine	1.23
1-palmitoyl-2-stearoyl-GPC (16:0/18:0)	1.23
threonine	1.23
N-acetylglucosaminylasparagine	1.23
glutarate (C5-DC)	1.23
oleoyl ethanolamide	1.22
furosemide	1.22
gamma-glutamyl-alpha-lysine	1.22
methylmalonate (MMA)	1.22
lactosyl-N-palmitoyl-sphingosine (d18:1/16:0)	1.21
3-hydroxyisobutyrate	1.21
N2-acetyl,N6-methyllysine	1.20
1-palmitoyl-GPE (16:0)	1.20

arabonate/xylonate	1.20
5-acetylamino-6-amino-3-methyluracil	1.20
3-bromo-5-chloro-2,6-dihydroxybenzoic acid*	1.20
catechol sulfate	1.19
N-acetylmethionine	1.19
N-methyltaurine	1.18
behenoyl sphingomyelin (d18:1/22:0)*	1.17
1-stearoyl-2-arachidonoyl-GPE (18:0/20:4)	1.17
bilirubin (Z,Z)	1.17
2-hydroxy-4-(methylthio)butanoic acid	1.17
3-methyl catechol sulfate (2)	1.17
1-linoleoyl-GPG (18:2)*	1.16
bilirubin (E,Z or Z,E)*	1.16
2,3-dihydroxy-5-methylthio-4-pentenoate (DMTPA)*	1.16
3-methyladipate	1.15
3-hydroxybutyroylglycine**	1.15
4-methylcatechol sulfate	1.15
eicosapentaenoylcholine	1.15
xanthurenate	1.15
S-methylmethionine	1.15
cysteine-glutathione disulfide	1.14
3-(3-amino-3-carboxypropyl)uridine*	1.14
tetrahydrocortisone glucuronide (5)	1.14
donepezil	1.14
2-methylcitrate/homocitrate	1.13
N-oleoylserine	1.13
10-undecenoate (11:1n1)	1.13
1-palmitoylglycerol (16:0)	1.13
vanillic alcohol sulfate	1.12
fibrinopeptide B (1-13)**	1.11
3-carboxy-4-methyl-5-propyl-2-furanpropanoate (CMPF)	1.10
1-stearoyl-GPE (18:0)	1.10
4-acetylcatechol sulfate (1)	1.10
1-(1-enyl-palmitoyl)-GPC (P-16:0)*	1.10
1-dihomo-linolenylglycerol (20:3)	1.10
sitagliptin	1.10
dimethyl sulfone	1.09
maleate	1.09

acesulfame	1.09
fibrinopeptide A (7-16)*	1.08
3,4-dihydroxybutyrate	1.08
gamma-glutamylphenylalanine	1.08
3-ethylcatechol sulfate (1)	1.08
5-oxoproline	1.08
benzoate	1.07
glycerophosphoglycerol	1.07
1-palmitoyl-2-arachidonoyl-GPE (16:0/20:4)*	1.07
palmitoylcholine	1.07
N6-acetyllysine	1.07
homovanillate (HVA)	1.07
ethyl beta-glucopyranoside	1.07
azelate (C9-DC)	1.07
alanine	1.07
dehydroepiandrosterone sulfate (DHEA-S)	1.06
succinoyltaurine	1.06
C-glycosyltryptophan	1.06
(2,4 or 2,5)-dimethylphenol sulfate	1.06
taurocholenate sulfate*	1.06
2-oxoarginine*	1.06
4-hydroxyglutamate	1.06
oxypurinol	1.06
citraconate/glutaconate	1.05

**Table S3.** differentially expressed metabolites **used to** evaluate the diagnostic efficacy. All metabolites met the following criteria: FC>1.5, FDR<0.05, and VIP (based on OPLSDA)>2
(14 or 15)-methylpalmitate (a17:0 or i17:0)
1-linoleoyl-GPA (18:2)*
1-oleoyl-GPE (18:1)
10-heptadecenoate (17:1n7)
11beta-hydroxyetiocholanolone glucuronide*
12-HHTrE
2-stearoyl-GPE (18:0)*
2,3-dihydroxy-2-methylbutyrate
5-dodecenoate (12:1n7)

5alpha-androstan-3alpha,17beta-diol monosulfate (1)
5alpha-androstan-3beta,17beta-diol monosulfate (2)
5alpha-pregnan-3beta,20beta-diol monosulfate (1)
androstenediol (3alpha, 17alpha) monosulfate (2)
bilirubin degradation product, C16H18N2O5 (3)**
bilirubin degradation product, C16H18N2O5 (4)**
cysteinylglycine
diacylglycerol (16:1/18:2 [2], 16:0/18:3 [1])*
dihomo-linolenoylcarnitine (C20:3n3 or 6)*
dopamine 3-O-sulfate
dopamine 4-sulfate
formiminoglutamate
hydantoin-5-propionate
isocaproate (i6:0)
isovalerate (i5:0)
L-urobilin
laurate (12:0)
linoleamide (18:2n6)
linolenamide (18:3)*
margaramide (17:0)*
myristamide (14:0)*
myristoleamide (14:1)*
myristoleate (14:1n5)
N-oleoyltaurine
N-palmitoylglycine
N-stearoyltaurine
octadecenedioylcarnitine (C18:1-DC)*
palmitamide (16:0)
palmitoleamide (16:1)*
palmitoleate (16:1n7)
phenylacetylglycine
pimelate (C7-DC)
sphinganine-1-phosphate

stearamide (18:0)
vanillactate

Table S4. ROC analysis, based on gender and age-adjusted logistic regression with 10- fold cross-validation, of individual metabolites in table S3. Only metabolites with p<0.05 are shown

**Metabolite name**	**AUC**	**Sensitivity**	**Specificity**	**Odds ratio**	**p-value**
(14 or 15)-methylpalmitate (a17:0 or i17:0)	0.879 (0.809 ~ 0.949)	0.818 (0.818 ~ 0.932)	0.889 (0.805 ~ 0.973)	0.04	<0.001
isovalerate (i5:0)	0.872 (0.800 ~ 0.944)	0.841 (0.841 ~ 0.949)	0.815 (0.711 ~ 0.918)	0.06	0.001
linolenamide (18:3)*	0.870 (0.796 ~ 0.943)	0.795 (0.795 ~ 0.915)	0.870 (0.781 ~ 0.960)	0.22	0.002
octadecenedioylcarnitine (C18:1-DC)*	0.866 (0.790 ~ 0.943)	0.750 (0.750 ~ 0.878)	0.926 (0.856 ~ 0.996)	0.06	< 0.001
palmitoleamide (16:1)*	0.856 (0.777 ~ 0.936)	0.818 (0.818 ~ 0.932)	0.870 (0.781 ~ 0.960)	0.33	0.002
1-linoleoyl-GPA (18:2)*	0.848 (0.767 ~ 0.928)	0.841 (0.841 ~ 0.949)	0.759 (0.645 ~ 0.873)	5.95	0.003
myristoleamide (14:1)*	0.847 (0.766 ~ 0.927)	0.909 (0.909 ~ 0.994)	0.722 (0.603 ~ 0.842)	0.4	0.001
sphinganine-1-phosphate	0.846 (0.760 ~ 0.931)	0.795 (0.795 ~ 0.915)	0.852 (0.757 ~ 0.947)	0.24	0.005
dihomo-linolenoylcarnitine (C20:3n3 or 6)*	0.843 (0.763 ~ 0.922)	0.727 (0.727 ~ 0.859)	0.815 (0.711 ~ 0.918)	0.2	0.002
bilirubin degradation product, C16H18N2O5 (4)**	0.843 (0.762 ~ 0.925)	0.750 (0.750 ~ 0.878)	0.889 (0.805 ~ 0.973)	0.4	0.05
N-oleoyltaurine	0.840 (0.756 ~ 0.925)	0.750 (0.750 ~ 0.878)	0.870 (0.781 ~ 0.960)	0.34	0.026
L-urobilin	0.839 (0.753 ~ 0.925)	0.818 (0.818 ~ 0.932)	0.833 (0.734 ~ 0.933)	3.03	0.01
diacylglycerol (16:1/18:2 [2], 16:0/18:3 [1])*	0.837 (0.749 ~ 0.925)	0.727 (0.727 ~ 0.859)	0.926 (0.856 ~ 0.996)	0.32	0.002
12-HHTrE	0.835 (0.754 ~ 0.916)	0.705 (0.705 ~ 0.839)	0.870 (0.781 ~ 0.960)	0.3	0.013
2-stearoyl-GPE (18:0)*	0.834 (0.750 ~ 0.918)	0.750 (0.750 ~ 0.878)	0.833 (0.734 ~ 0.933)	3.75	0.014
N-palmitoylglycine	0.834 (0.749 ~ 0.920)	0.795 (0.795 ~ 0.915)	0.815 (0.711 ~ 0.918)	0.15	0.024
myristamide (14:0)*	0.829 (0.745 ~ 0.912)	0.727 (0.727 ~ 0.859)	0.870 (0.781 ~ 0.960)	0.3	0.024
cysteinylglycine	0.828 (0.742 ~ 0.915)	0.818 (0.818 ~ 0.932)	0.796 (0.689 ~ 0.904)	0.26	0.01
isocaproate (i6:0)	0.824 (0.736 ~ 0.912)	0.795 (0.795 ~ 0.915)	0.833 (0.734 ~ 0.933)	0.27	0.02
N-stearoyltaurine	0.819 (0.726 ~ 0.911)	0.773 (0.773 ~ 0.897)	0.833 (0.734 ~ 0.933)	0.35	0.042
